# Signature-Based Small Molecule Screening Identifies Cytosine Arabinoside as an EWS/FLI Modulator in Ewing Sarcoma

**DOI:** 10.1371/journal.pmed.0040122

**Published:** 2007-04-10

**Authors:** Kimberly Stegmaier, Jenny S Wong, Kenneth N Ross, Kwan T Chow, David Peck, Renee D Wright, Stephen L Lessnick, Andrew L Kung, Todd R Golub

**Affiliations:** 1 Department of Pediatric Oncology, Dana-Farber Cancer Institute and Children's Hospital Boston, Harvard Medical School, Boston, Massachusetts, United States of America; 2 The Broad Institute of Harvard University and Massachusetts Institute of Technology, Cambridge, Massachusetts, United States of America; 3 Howard Hughes Medical Institute, Harvard Medical School, Boston, Massachusetts, United States of America; 4 The Center for Children, Huntsman Cancer Institute, University of Utah, Salt Lake City, Utah, United States of America; National Cancer Institute, United States of America

## Abstract

**Background:**

The presence of tumor-specific mutations in the cancer genome represents a potential opportunity for pharmacologic intervention to therapeutic benefit. Unfortunately, many classes of oncoproteins (e.g., transcription factors) are not amenable to conventional small-molecule screening. Despite the identification of tumor-specific somatic mutations, most cancer therapy still utilizes nonspecific, cytotoxic drugs. One illustrative example is the treatment of Ewing sarcoma. Although the EWS/FLI oncoprotein, present in the vast majority of Ewing tumors, was characterized over ten years ago, it has never been exploited as a target of therapy. Previously, this target has been intractable to modulation with traditional small-molecule library screening approaches. Here we describe a gene expression–based approach to identify compounds that induce a signature of EWS/FLI attenuation. We hypothesize that screening small-molecule libraries highly enriched for FDA-approved drugs will provide a more rapid path to clinical application.

**Methods and Findings:**

A gene expression signature for the EWS/FLI *off* state was determined with microarray expression profiling of Ewing sarcoma cell lines with EWS/FLI-directed RNA interference. A small-molecule library enriched for FDA-approved drugs was screened with a high-throughput, ligation-mediated amplification assay with a fluorescent, bead-based detection. Screening identified cytosine arabinoside (ARA-C) as a modulator of EWS/FLI. ARA-C reduced EWS/FLI protein abundance and accordingly diminished cell viability and transformation and abrogated tumor growth in a xenograft model. Given the poor outcomes of many patients with Ewing sarcoma and the well-established ARA-C safety profile, clinical trials testing ARA-C are warranted.

**Conclusions:**

We demonstrate that a gene expression–based approach to small-molecule library screening can identify, for rapid clinical testing, candidate drugs that modulate previously intractable targets. Furthermore, this is a generic approach that can, in principle, be applied to the identification of modulators of any tumor-associated oncoprotein in the rare pediatric malignancies, but also in the more common adult cancers.

## Abstract 2

Todd Golub and colleagues show that a gene expression-based screen of small-molecule libraries can identify candidate drugs that modulate cancer-associated oncoproteins.

## Editors' Summary

**Background.:**

Cancer occurs when cells accumulate genetic changes (mutations) that allow them to divide uncontrollably and to travel throughout the body (metastasize). Chemotherapy, a mainstay of cancer treatments, works by killing rapidly dividing cells. Because some normal tissues also contain dividing cells and are therefore sensitive to chemotherapy drugs, it is hard to treat cancer without causing serious side effects. In recent years, however, researchers have identified some of the mutations that drive the growth of cancer cells. This raises the possibility of designing drugs that kill only cancer cells by specifically targeting “oncoproteins” (the abnormal proteins generated by mutations that transform normal cells into cancer cells). Some “targeted” drugs have already reached the clinic, but unfortunately medicinal chemists do not know how to inhibit the function of many classes of oncoproteins with the small organic molecules that make the best medicines. One oncoprotein in this category is EWS/FLI. This contains part of a protein called EWS fused to part of a transcription factor (a protein that controls cell behavior by telling the cell which proteins to make) called FLI. About 80% of patients with Ewing sarcoma (the second commonest childhood cancer of bone and soft tissue) have the mutation responsible for EWS/FLI expression. Localized Ewing sarcoma can be treated with nontargeted chemotherapy (often in combination with surgery and radiotherapy), but treatment for recurrent or metastatic disease remains very poor.

**Why Was This Study Done?:**

Researchers have known for years that EWS/FLI expression drives the development of Ewing sarcoma by activating the expression of target genes needed for tumor formation. However, EWS/FLI has never been exploited as a target for therapy of this cancer—mainly because traditional approaches used to screen libraries of small molecules do not identify compounds that modulate the activity of transcription factors. In this study, the researchers have used a new gene expression–based, high-throughput screening (GE-HTS) approach to identify compounds that modulate the activity of EWS/FLI.

**What Did the Researchers Do and Find?:**

The researchers used a molecular biology technique called microarray expression profiling to define a 14-gene expression signature that differentiates between Ewing sarcoma cells in which the EWS/FLI fusion protein is active and those in which it is inactive. They then used this signature to screen a library of about 1,000 chemicals (many already approved for other clinical uses) in a “ligation-mediated amplification assay.” For this, the researchers grew Ewing sarcoma cells with the test chemicals, extracted RNA from the cells, and generated a DNA copy of the RNA. They then added two short pieces of DNA (probes) specific for each signature gene to the samples. In samples that expressed a given signature gene, both probes bound and were then ligated (joined together) and amplified. Because one of each probe pair also contained a unique “capture sequence,” the signature genes expressed in each sample were finally identified by adding colored fluorescent beads, each linked to DNA complementary to a different capture sequence. The most active modulator of EWS/FLI activity identified by this GE-HTS approach was cytosine arabinoside (ARA-C). At levels achievable in people, this compound reduced the abundance of EWS/FLI protein in and the viability and cancer-like behavior of Ewing sarcoma cells growing in test tubes. ARA-C treatment also slowed the growth of Ewing sarcoma cells transplanted into mice.

**What Do These Findings Mean?:**

These findings identify ARA-C, which is already used to treat children with some forms of leukemia, as a potent modulator of EWS/FLI activity. More laboratory experiments are needed to discover how ARA-C changes the behavior of Ewing sarcoma cells. Nevertheless, given the poor outcomes currently seen in many patients with Ewing sarcoma and the historical reluctance to test new drugs in children, these findings strongly support the initiation of clinical trials of ARA-C in children with Ewing sarcoma. These results also show that the GE-HTS approach is a powerful way to identify candidate drugs able to modulate the activity of some of the oncoproteins (including transcription factors and other previously intractable targets) that drive cancer development.

**Additional Information.:**

Please access these Web sites via the online version of this summary at http://dx.doi.org/10.1371/journal.pmed.0040122.

- Cancerquest from Emory University, provides information on cancer biology (also includes information in Spanish, Chinese and Russian)
- The MedlinePlus encyclopedia has pages on Ewing sarcoma
- Information for patients and health professionals on Ewing sarcoma is available from the US National Cancer Institute
- Cancerbackup offers information for patients and their parents on Ewing sarcoma
- Wikipedia has pages on DNA microarrays and expression profiling (note that Wikipedia is a free online encyclopedia that anyone can edit)

## Introduction

A major challenge in pediatric oncology is the timely development and testing of drugs for children with cancer. Because the pediatric malignancies are rare, they are not an industry priority for drug development. In addition, there is a historical reluctance to test new drugs in children. In fact, almost all therapies currently used in the treatment of pediatric cancers were first tested in adults. Despite this lack of progress in treatment, significant advances have been made in understanding the molecular basis of pediatric malignancies. Frequently, these tumors are associated with tumor-specific somatic mutations generating oncoproteins [1–3]. In principle, these oncoproteins represent unique, tumor cell–specific targets for therapeutic development. In practice, however, few oncoprotein-targeted drugs, particularly for the pediatric malignancies, have entered clinical trial. Here lies a second challenge. Many of the identified oncoproteins present in pediatric tumors, particularly those involving transcription factors, are considered “undruggable.” These targets have been largely ignored because there is no obvious approach to pharmacologically inhibiting their function. Traditional, biochemical small-molecule screens might be directed against druggable downstream effectors of these oncoproteins, but in most cases, such critical effectors have yet to be identified.

The pediatric solid tumor Ewing sarcoma is one example of these challenges. Ewing sarcoma treatment exemplifies the problem of molecularly informed therapy lagging behind identification of a pathognomonic genetic lesion. Over ten years ago, the translocation t(11;22)(q24;q12) was characterized and subsequently identified in over 80% of patients with Ewing sarcoma, yet no oncoprotein-directed therapy has been developed [2,4]. This translocation fuses the amino terminus of the EWS protein to the DNA-binding domain of the ETS transcription factor FLI. The resultant EWS/FLI fusion protein is believed to function by transcriptionally dysregulating target genes critical for tumor formation [5–7].

EWS/FLI is an attractive treatment target for Ewing sarcoma because of its malignant cell specificity. Furthermore, experimental evidence indicates that EWS/FLI expression is essential for Ewing sarcoma tumor cells. In vitro targeting of EWS/FLI with antisense oligodeoxynucleotides and RNA interference (RNAi) inhibits Ewing sarcoma cell viability, growth, and oncogenic transformation, supporting EWS/FLI attenuation as a potential treatment modality [8–14]. However, to date, Ewing therapy has largely consisted of combinations of nontargeted cytotoxic agents. Event-free survival at five years for patients with metastatic disease is only 20% [15,16], and curative therapy does not yet exist for patients whose disease recurs rapidly following therapy for localized disease.

To overcome the current limitations to small-molecule library screening, we developed gene expression-based high-throughput screening (GE-HTS) [17]. GE-HTS uses gene expression signatures as surrogates for cellular states. First, gene expression–based signatures of the biological states of interest (i.e., “state A” versus “state B”) are defined using genome-wide gene expression profiling. Next, an assay is developed (as described below) for the high-throughput, low-cost measurement of the signature. Then, a small-molecule library is screened for compounds that induce a change from the “state A” to the “state B” signature. Unlike traditional phenotype-based screens, GE-HTS does not require development of specialized assays. The gene expression signature definition, amplification, and detection are generic. Furthermore, a priori knowledge of a target is not needed. The gene expression signature serves as a surrogate for the biological state in question. The method is thus well suited for discovery of modulators of oncogenic transcription factors in which the mutant transcription factor's transforming mechanism is not known.

We hypothesized that drugs may already exist for the treatment of Ewing sarcoma, but tools to prioritize candidates for clinical testing have been lacking. We therefore performed a GE-HTS-based small-molecule library screen to identify modulators of EWS/FLI activity in Ewing sarcoma and focused our efforts on collections of bioactive compounds that contain FDA-approved drugs.

## Methods

### Cell Culture

The Ewing sarcoma cell lines A673, EWS502, and TC71 were grown as previously described [18]. The EWS502 line was originally developed by Jonathan Fletcher's laboratory and is reported to have a type 1 EWS/FLI fusion (J. Fletcher, personal communication). Its growth arrests in response to EWS/FLI knockdown and requires both NKX2.2 and NR0B1 for growth and transformation [14,19]. EWS502 harbors the previously reported C135F mutation in the tumor suppressor p53 [20,21]. Tet-A673 cells harboring an inducible *EWS/FLI* cDNA were also previously described [14]. TC32 Ewing sarcoma cells were grown in RPMI 1640 (Cellgro, http://www.cellgro.com) with 10% FBS (Sigma,  http://www.sigmaaldrich.com), penicillin-streptomycin (Cellgro), and glutamine (Gibco, http://www.invitrogen.com). The carcinoma cell lines A549, HCT116, SW480, and T47D were all purchased from the American Type Culture Collection (ATCC, http://www.atcc.org) and grown in Ham's F-10 (A549) (Cellgro), RPMI 1640 (HCT116), and DMEM (SW480 and T47D) (Cellgro) supplemented with penicillin-streptomycin and 10% FBS. HL-60 and U937 cells were purchased from ATCC and grown in RPMI 1640 with 10% FBS and 1% penicillin-streptomycin.

Growth analysis using a serial passage 3T10 assay was performed by plating 1 × 10^6^ cells in a 10-cm tissue culture dish every 3 d and counting the resultant number of cells 3 d later over 15 d. In some samples, 30 nM or 60 nM cytosine arabinoside (ARA-C) was included in the culture medium.

Soft agar assays were performed essentially as previously described, except that 30 nM or 60 nM ARA-C was included in the agar layers and medium in some samples [18].

### Retroviruses

The retroviral RNAi vectors EF-2-RNAi and luc-RNAi were previously described [14]. Retroviral production and infection were performed as previously described [18]. Following retroviral infection, cells were selected and maintained in 2 μg/ml of puromycin (Sigma).

### Immunodetection

Cells were lysed in RIPA buffer (10 mM Tris-Cl [pH 7.6], 100 mM NaCl, 1 mM EDTA, 1% Triton X-100, 0.5% sodium deoxycholate, and 0.1% SDS) with protease inhibitor (Complete Mini EDTA free protease inhibitor tablets, Roche Diagnostics, http://www.roche.com), resolved by electrophoresis on 10% Tris-HCl precast Ready Gel (BioRad Laboratories, http://www.bio-rad.com), and transferred to nitrocellulose membranes (Schleicher and Schuell, http://www.schleicher-schuell.com) for whole cell lysates. All proteins were detected using chemiluminescence and antibodies to FLI-1 (sc-356, Santa Cruz Biotechnology, http://www.scbt.com), pan-actin antibody (ACTN05, NeoMarkers, http://www.labvision.com), acetyl-histone H3 (06–599, Upstate Cell Signaling Solutions, http://www.upstate.com), Sp3 (07–107, Upstate Cell Signaling Solutions), BTF3 (38–6100, Zymed Laboratories, Invitrogen Immunodetection), and YY1 (AP2517a, Abgent, http://www.abgent.com).

### Marker Gene Selection

The Affymetrix U133A datasets used for marker gene identification were previously reported [14] and are available at http://www.broad.mit.edu/cancer/Ewing_ARA-C. The first dataset was derived from A673 Ewing sarcoma cells expressing one of two different EWS/FLI retroviral knockdown constructs (EF-2-RNAi or EF-4-RNAi, herein called “EWS/FLI-knockdown”) or a control construct directed against luciferase (herein called “EWS/FLI-expressing”). GeneChip MAS5 software (Affymetrix, http://www.affymetrix.com) was used for preprocessing of the raw data, and all scans within an experiment were scaled to the array with the median overall microarray intensity. Thresholds were set to a minimum value of 10 and a maximum value of 16,000, and a variation filter of 5-fold minimum and 50 minimum absolute difference was applied. We used the signal-to-noise ratio to rank order the genes that distinguish “EWS/FLI-expressing” from “EWS/FLI-knockdown” and then permutation testing to identify genes that distinguish these states with a *p* < 0.05 [22]. We next identified genes with at least a 2.5-fold difference between the two classes of interest and an expression value of less than 25 in one of the two states. We then evaluated these genes in an EWS/FLI-inducible rescue dataset. This dataset was generated from Tet-A673 inducible Ewing sarcoma cells expressing the EF-2-RNAi construct to knock down endogenous EWS/FLI expression and subsequently induced to express the exogenous *EWS/FLI* cDNA. Data points were collected at various times after induction. Through a comparison of the stable knockdown and inducible rescue datasets, we selected 14 genes that were well correlated with EWS/FLI expression in both datasets. We included *glyceraldehyde-3-phosphate dehydrogenase (GAPD)* as a control for well-to-well variability, as it demonstrated a stable expression pattern in all conditions. We also included a probe for FLI1 to monitor effective knockdown in the positive control wells with EWS/FLI-directed retroviral RNAi constructs.

### Small-Molecule Library Screening

#### Cell and compound addition.

A673 cells were plated in 384-well tissue culture plates in 50 μl of medium at 6,000 cells/well. The EWS/FLI short hairpin RNA (shRNA) infected A673 controls (EF-2-RNAi) were grown at 8,000 cells/well. The following controls were included on each 384-well screen plate: medium only (16 wells), A673 cells with DMSO vehicle (eight wells), and EF-2-RNAi (eight wells). In addition, two plates with controls exclusively were included in the screen: medium (44 wells), A673 + DMSO (154 wells), and EF-2-RNAi (154 wells).

Compounds were added at a final approximate concentration of 20 μM in DMSO and incubated for 3 d at 37 °C with 5% CO_2._ We screened in triplicate the National Institute of Neurological Disorders and Stroke small-molecule collection containing 1,040 compounds (http://www.broad.harvard.edu/chembio/platform/screening/compound_libraries/ninds.htm). A total of three-quarters of the compounds in this collection of characterized bioactive molecules are FDA approved.

#### RNA extraction and reverse transcription.

After 3 d of chemical incubation, 40 μl of medium was removed from the wells with a Multimek robot (Beckman Coulter, http://www.beckmancoulter.com). Cells were then lysed with 25 μl of lysis buffer for 30 min at room temperature. Lysate was transferred to a 384-well oligo-dT coated plate and incubated for 1 h at room temperature. Reverse transcription was carried out in a 5-μl MMLV reaction (Pierce, http://www.piercenet.com) for 1.5 h at 37 °C. Lysis buffers and 384-well oligo-dT plates were purchased from RNAture (Hitachi Chemical, http://www1.qiagen.com).

#### Ligation-mediated amplification.

After 1.5 h, unbound material was removed by inverting the plate onto an absorbent towel and spinning in the centrifuge. Signature gene-specific oligonucleotide probes were hybridized to the cDNA using 2 nM of each probe (16 probe pairs in total) and Taq ligase buffer (New England Biolabs, http://www.neb.com) in a 5-μl reaction. Upstream probes contained the T7 primer site, Luminex-designed FlexMap barcode tag (24 nt), and gene-specific sequence (20 nt). Downstream probes were phosphorylated at the 5′ end and contained gene-specific sequence (20 nt) followed by the T3 primer site. Gene-specific sequence was chosen such that the 20-base-pair sequences of the upstream and downstream probes contain similar base composition, minimal repeats, and C-G or G-C juxtaposing nucleotides (Table S1). Hybridization was performed at 95 °C for 2 min followed by 50 °C for 1 h. Excess probe was then spun out. Probes were ligated in a 5-μl reaction using Taq DNA ligase (New England Biolabs) at 45 °C for 1 h followed by incubation at 65 °C for 10 min. Excess ligation mix was spun out. The ligated products were amplified with the universal T3 primer (5′-ATT AAC CCT CAC TAA AGG GA-3′) and universal biotinylated T7 primer (5′-TAA TAC GAC TCA CTA TAG GG-3′) (Integrated DNA Technologies, http://www.idtdna.com) using HotStarTaq DNA Polymerase (Qiagen, http://www1.qiagen.com) in a 7-μl reaction system. PCR was performed at 92 °C for 9 min, followed by 39 cycles of denaturation at 92 °C for 30 s, annealing at 60 °C for 30 s, and extension at 72 °C for 30 s.

#### Amplicon detection.

xMAP Multi-Analyte COOH microspheres (Luminex, http://www.luminexcorp.com) (2.5 million) were coupled to complementary FlexMap barcode sequence (4 μM) with 2.5 μl of 10 mg/ml 1-ethyl-3-(3-dimethylaminopropyl)-carbodiimide hydrochloride (EDC) (Pierce) in 25 μl 0.1 M 2-(*N*-morpholino) ethanesulfonic acid (pH 4.5) (MES) buffer (Sigma). This 30-min coupling reaction was then repeated. Microspheres were then washed sequentially with 0.02% Tween-20, 0.1% SDS, and TE (pH 8), and resuspended in 50 μl of TE (pH 8). Next, a ligation-mediated amplification (LMA) sample was hybridized to microspheres by incubating 2,500 of each microsphere in 18 μl of 1.5× TMAC (4.5 M tetramethylammonium chloride, 0.15% *N*-lauryl sarcosine, 75 mM Tris-HCl [pH 8], and 6 mM EDTA [pH 8]) and 5 μl of TE (pH 8) at 95 °C for 2 min and then 45 °C for 1 h. For detection, the sample was incubated with 10 μl of streptavidin-phycoerythrin (SA-PE) (Invitrogen) (1% SA-PE in 1× TMAC [3 M tetramethylammonium chloride], 0.1% *N*-lauryl sarcosine, 50 mM Tris-HCl [pH 8], and 4 mM EDTA [pH 8]) for 5 min at 45 °C and then washed once and resuspended with 1× TMAC. Dual-color fluorescence was detected with a Luminex high-throughput detection instrument. A minimum of 100 events was recorded for each microsphere, and median fluorescent intensities computed.

#### Hit identification.

The median fluorescence level for each gene (where each gene was represented by a particular bead color) from the Luminex detector was used as a measure of the gene's expression. To maximize consistency between and within plates, we normalized genes by using the expression ratio between each gene and *GAPD*. Next, filtering was performed to eliminate “dead” wells from further analysis where *GAPD* expression levels were used as a proxy for cell viability. Because ratios with *GAPD* as the denominator were used for subsequent analysis, we needed to eliminate wells where *GAPD* was nominally zero. Forming a ratio with such a low denominator could potentially falsely flag a well where the cells were merely dead or dying. For our filtering level, we used the mean minus two times the standard deviation of the untreated A673 control wells. For this screen, 128 out of 3,840 compound wells were filtered. After filtering, we used a “summed score” metric to identify candidate modulators of EWS/FLI. This metric combined expression ratios by summing them with a sign determined by the expected direction of regulation as determined from the EWS/FLI shRNA samples. Compounds were ranked for follow-up according to the total of the summed score from the three replicates.

### Evaluation of Whole Genome Effects

#### Gene set enrichment analysis.

To test ARA-C's affects on the EWS/FLI expression program, an expanded signature for EWS/FLI knockdown (Table S2) was created from the EWS/FLI RNAi dataset and tested for enrichment using the gene set enrichment analysis (GSEA) method with publicly available software (http://www.broad.mit.edu/gsea) [23]. A gene set for EWS/FLI knockdown was created by thresholding (10 minimum and 16,000 maximum) and filtering (5-fold minimum variation and 50 minimum absolute difference), which resulted in 1,460 genes that passed filtering. All genes that distinguished EWS/FLI shRNA versus the luciferase controls (t-test *p* < 0.01 [74 genes up-regulated and 93 genes down-regulated with EWS/FLI knockdown]) and confirmed to have a consistent direction of change in the inducible EWS/FLI dataset (66 genes up-regulated and 77 genes down-regulated with EWS/FLI knockdown) were identified. The 66-gene EWS/FLI up-regulated gene set was tested for enrichment in sample sets formed out of the 17 DMSO-treated A673 samples versus the combined 3-d and 5-d compound-treated sets (typically six samples each for ARA-C, doxorubicin, and puromycin). The enrichment of the members of the EWS/FLI gene set was measured by the enrichment score, and statistical significance was determined using permutation testing (2,500 permutations). The results of the GSEA are summarized in Table S3. Sample and cel file names are listed in Table S4. Raw data are available at http://www.broad.mit.edu/cancer/Ewing_ARA-C or at GEO (Gene Expression Omnibus, http://www.ncbi.nlm.nih.gov/projects/geo/query/acc.cgi) series record GSE6930.

#### Sample preparation.

RNA was extracted with Trizol per the manufacturer's protocol (Invitrogen) and prepared for hybridization to Affymetrix high-throughput arrays. We used 2 μg of RNA for target preparation as per the Affymetrix Gene Chip Automated Station (GCAS) protocol (Early Access User manual version 1.0) and 3.5 μg of cRNA hybridized to Affymetrix 96-well Gene Chip HT arrays.

### Viability Assay

Viability experiments were performed in 96-well format in replicates of four using the Promega Cell-Titer Glo ATP-based assay per the manufacturer's instructions (http://www.promega.com). Cells were evaluated at 0, 3, and 6 d with ARA-C in a 2-fold dilution from 10 μM down to 78 nM versus water-control–treated cells. The concentration at which cell viability was reduced to 50% of water-treated controls (EC_50_) was determined. Values for EC_50_ were calculated by interpolating a polynomial fit to the measured viability data. Curve fitting was performed in MATLAB (http://www.mathworks.com) using the least-squares curve-fitting function (polyfit). Model order for the polynomial was set to *n* = 3. The EC_50_ value was found by interpolating the curve for the value of 50 with the MATLAB one-dimensional interpolation function (interp1).

### Real-Time Reverse Transcriptase PCR

Total RNA was isolated using TRIZOL reagent (Invitrogen) from A673 cells treated with vehicle, ARA-C 170 nM, and ARA-C 340 nM for 3 d. cDNA was synthesized from 1 μg of total RNA using SuperScript III Reverse Transcriptase (Invitrogen) and oligo d(T)_16_ primers in a 20-μl reaction system. cDNA (1 μl) was analyzed in the real-time quantitative PCR reactions prepared with TaqMan Universal Master mix (Applied Biosystems, http://www.appliedbiosystems.com). Each sample was assessed in triplicate to ensure reproducibility of the quantitative measurements. *GAPD* expression was evaluated for each sample as a control for total RNA. Primers and probes for real-time reverse transcriptase PCR were obtained from Applied Biosystems Assays: number 302414 for *FLI* and number 402869 for *GAPD*.

### Apoptosis Analysis

We cultured 1.5 × 10^6^ cells with and without ARA-C at the following concentrations, 170 nM, 340 nM, and 680 nM ARA-C. As a control for apoptosis, cells were also treated with 3.7 μM puromycin. Cells were treated for 3 d. Annexin V FITC/PI staining was performed using the Annexin V: FITC Apoptosis Detection Kit I (BD Pharmingen, http://www.bdbiosciences.com). Cells were analyzed by flow cytometry with a FACScan flow cytometer (Becton Dickinson, http://www.bd.com) and CELLQuest analytical software.

### Xenograft Studies

A673 cells were infected with retroviruses expressing a luciferase–neomycin resistance gene fusion protein and selected with G418 [14]. Following selection, 1 × 10^6^ cells were injected into the flanks of NCr nude mice, and A673-luciferase–positive xenografts were established for 2–3 wk with tumor burden monitored by bioluminescence imaging. Mice with logarithmically growing tumors were treated with intraperitoneal injection of ARA-C at 50 mg/kg daily for four doses or vehicle control. We treated nine mice per condition. Mice were imaged weekly using a Xenogen IVIS 100 imaging system, per the manufacturer's directions (http://www.xenogen.com). Student's t-test (two-tailed assuming samples with equal variance) was used to compare tumor burden across the two cohorts. Animal experiments were performed following approval from the Dana-Farber Cancer Institute Animal Care and Use Committee (http://www.dfci.harvard.edu).

## Results

### EWS/FLI *Off* Marker Gene Signature Identified for GE-HTS

The GE-HTS screening approach enables identification of small molecules that induce a change in expression signature from the EWS/FLI *on* state to the EWS/FLI *off* state. We first needed to design a signature of EWS/FLI loss of function. Accordingly, we established the transcriptional profile of A673 Ewing sarcoma cells in the presence or absence of an shRNA directed against the endogenous EWS/FLI transcript, as described in detail elsewhere [14]. A673 cells were chosen because knockdown of EWS/FLI results in loss of tumorigenicity in soft agar and murine xenografts, but only minimally alters their normal growth in tissue culture. As such, the EWS/FLI knockdown signature is more likely to reflect EWS/FLI loss of function, rather than a general signature of growth arrest or apoptosis, as is observed in other Ewing cell lines [14]. A second challenge was controlling for the off-target effects of shRNA in the definition of an EWS/FLI signature. To address this, we used an EWS/FLI rescue system in which loss of oncogenic transformation with shRNA could be rescued by ectopic expression of an *EWS/FLI* cDNA lacking the 3′ UTR to which the EWS/FLI shRNA was directed [14].

**Figure 1:**
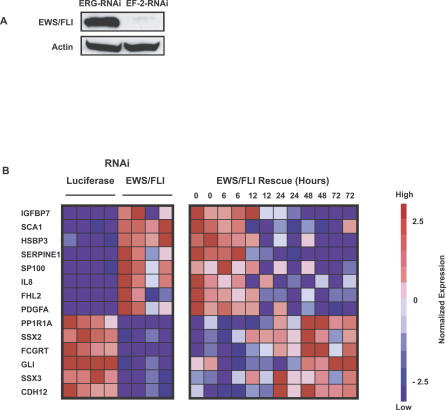
EWS/FLI *Off* Marker Gene Selection (A) EWS/FLI Western blot analysis with anti-FLI antibody confirms reduction in protein levels with infection of the A673 Ewing sarcoma cell line with retrovirus containing an shRNA construct directed against EWS/FLI (EF-2-RNAi) compared to a control with RNAi directed against EWS/ERG (ERG-RNAi) which is not expressed in A673 cells. Actin is shown as a loading control. (B) Gene expression profiling was performed with shRNA designed against a luciferase control (four replicates) and in duplicate against two different shRNA constructs against EWS/FLI. Using the signal-to-noise ratio, the genes distinguishing luciferase from EWS/FLI directed RNAi were identified and then prioritized for a fold change of at least 2.5 and a baseline level of expression less than 25 in one of the states. These genes were then evaluated in an inducible EWS/FLI rescue system. Exogenous EWS/FLI was induced over 72 h in A673 cells with EWS/FLI knockdown by shRNA, and samples evaluated in duplicate at multiple time points. The EWS/FLI *off* signature genes are shown in the heat map. Samples are shown in columns and genes in rows. Blue represents poorly expressed genes, and red depicts highly expressed genes.

We first designed a signature for use in the GE-HTS platform. Signature genes were selected based on the following criteria: (i) statistically different expression in the EWS/FLI knockdown versus control A673 cells (at *p* < 0.05 based on permutation testing); (ii) fold-change over 2.5; (iii) nearly undetectable expression in one of the two conditions; and (iv) reversion of expression with induction of the EWS/FLI rescue construct. We selected 14 genes meeting these criteria (Figure 1). *GAPD* was included as a control for well-to-well variability in the screen because of its stable expression across the dataset. FLI1 was included to demonstrate EWS/FLI knockdown in positive control wells with EWS/FLI directed shRNA. The final signature thus constituted 16 genes.

### GE-HTS Overview: LMA with Fluorescent Bead Detection

**Figure 2:**
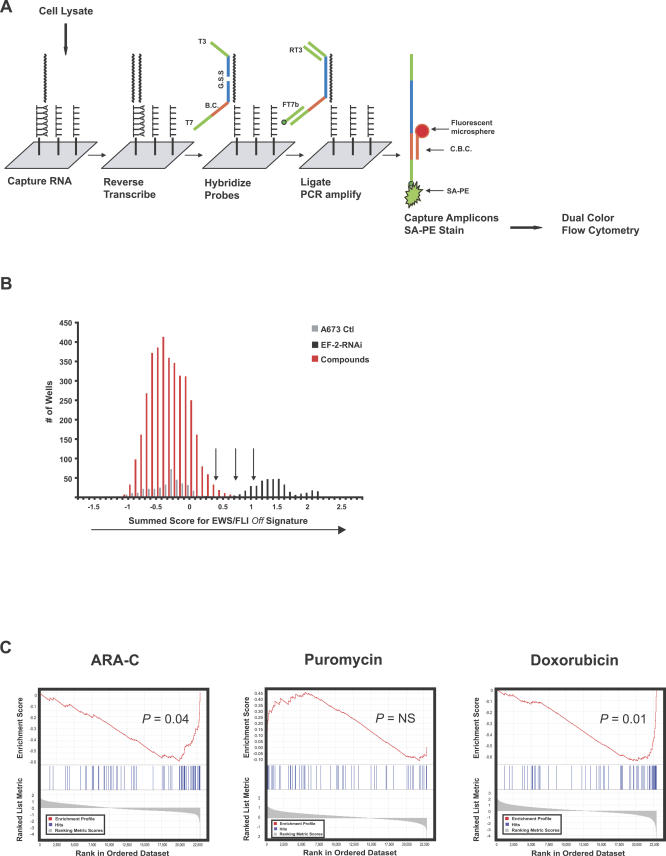
GE-HTS Identifies ARA-C as the Top Hit Inducing an EWF/FLI *Off* Signature (A) GE-HTS utilizes LMA with fluorescent microsphere detection of PCR amplicons. Cells are lysed, and lysate transferred to a 384-well plate coated in oligo-dT. Reverse transcription is performed and then probes hybridized to the cDNA. The upstream probe contains a universal T7 sequence, barcode capture sequence (B.C.), and gene-specific sequence (G.S.S.). The downstream probe contains gene-specific sequence and T3 universal sequence. The probes are then ligated and PCR performed with a universal, biotinylated T7 primer (FT7b) and a universal T3 primer (RT3). Amplicons are captured with fluorescent microspheres coupled to capture probes containing sequence complementary to the barcode capture sequence (C.B.C). Amplicons are stained with streptavidin-phycoerythrin (SA-PE) and then quantified with dual color flow cytometry. Bead color is used to identify each gene, and the amount of phycoerythrin fluorescence measures the quantity of transcript. (B) Distribution of the summed score for the EWS/FLI *off* marker genes is shown. Gray indicates the distribution for the A673 vehicle control samples, black indicates the distribution for the EWS/FLI shRNA controls (EF-2-RNAi), and red for the 1,040 compounds screened in triplicate. The black arrows indicate the location of each of the replicates of ARA-C treatment within the distribution. (C) GSEA results for the set of EWS/FLI shRNA up-regulated genes confirmed in the inducible EWS/FLI dataset (66 genes) tested at 3 and 5 d at the EC_50_ for ARA-C (170 nM), puromycin (90 nM), and doxorubicin (60 nM). The running enrichment score, generated by the markers after they have been ordered with the phenotype of interest, is shown in red. The position of the genes in the gene set is shown as a blue line (“hits”). The ranking scores for the genes are shown in gray. The amount of enrichment is estimated by the maximum deviation from zero of the enrichment score, and statistical significance was determined using permutation testing (2,500 permutations).

To measure the EWS/FLI signature in the setting of a small-molecule screen, we converted the 16-gene signature to a low-cost, high-throughput format. Our previous implementation of the GE-HTS method involved multiplexed reverse transcriptase-PCR followed by detection by mass spectrometry [17]. That method, however, is limited to signatures of approximately six genes. To overcome this limitation, we developed a new approach in which multiplexed LMA products are detected by bead-based flow cytometry. The LMA/bead-based detection protocol is described in detail elsewhere [24–26]. Briefly, mRNAs are captured on oligo-dT coated 384-well plates and reverse transcribed to generate first strand cDNA. Locus-specific oligonucleotides (oligos) corresponding to the 16 signature genes are then annealed to the cDNA template. The oligos are designed such that (i) the primer pairs abut each other, thereby facilitating subsequent ligation, (ii) one of the oligos incorporates a unique barcode sequence facilitating subsequent bead capture, and (iii) both oligos incorporate a common flanking sequence facilitating PCR amplification using a single set of PCR primers. One of the PCR primers is biotinylated to facilitate detection (Figure 2A). This amplification strategy enables the simultaneous amplification of the signature transcripts in a highly reproducible manner that is faithful to the abundance of the starting mRNAs [26]. Following PCR amplification, the LMA products are hybridized to beads coupled to capture probes complementary to the LMA barcode sequence. Then, staining with streptavidin–phycoerythrin is performed. The use of beads of 16 different colors (each corresponding to a different barcode capture probe) facilitates subsequent quantitation of the LMA products by flow cytometry. The color of the bead denotes the identity of the signature transcript, and the phycoerythrin intensity denotes the transcript's abundance (Figure 2A).

### ARA-C Identified as Top Hit in GE-HTS for Modulators of EWS/FLI

We screened in triplicate the National Institute of Neurological Disorders and Stroke bioactive small-molecule library containing 1,040 small molecules, including many FDA-approved drugs. A673 cells were incubated with one compound per well for three days. A marker gene summary score was calculated for each of the small molecules in the screen and compared to that of the shRNA directed against EWS/FLI versus parental A673 cells. ARA-C, a nucleoside analog known to inhibit DNA synthesis, was identified as the top hit scoring highly in all three replicates (Figure 2B). Repeat evaluation of the marker genes in A673 cells after ARA-C treatment confirmed induction of the EWS/FLI *off* signature (Figure S1). The library did not contain compounds such as vincristine, doxorubicin, or ifosfamide, agents currently used in the treatment of Ewing sarcoma. However, it did contain 22 cytotoxic agents, none of which scored as positives in the screen. Importantly, ARA-C did not elicit a “pseudo” EWS/FLI *off* signature in ARA-C-responsive, EWS/FLI-negative, acute myeloblastic leukemia (AML) cell lines (Figure S2), indicating that all cells exposed to ARA-C do not nonspecifically regulate the EWS/FLI signature.

### ARA-C Induces Whole-Genome Changes Consistent with EWS/FLI Knockdown

To determine whether ARA-C induces a whole genome program consistent with EWS/FLI attenuation, or merely alters the expression of the marker genes selected for the EWS/FLI *off* signature, we evaluated genome-wide expression changes associated with ARA-C using GSEA [23]. GSEA is a nonparametric analytical approach used to determine whether the members of a set of genes of interest (e.g., genes associated with EWS/FLI *off*) tend to occur toward the top of a ranked list of genes corresponding to a class distinction (e.g., vehicle-treated versus ARA-C-treated A673 cells). In other words, is the list of genes that distinguish vehicle- versus ARA-C-treated A673 cells enriched for those genes whose expression changes with knockdown of EWS/FLI? We expected gene sets that are highly correlated with this class distinction to be found at the top of such a list and those unrelated to be randomly distributed throughout. Our EWS/FLI shRNA dataset was used to create an expanded list of EWS/FLI *off* marker genes (Table S2). A total of 143 genes (66 up-regulated and 77 down-regulated) distinguished EWS/FLI shRNA from the luciferase controls (*p* < 0.01) and were at least partially reverted by the EWS/FLI rescue construct. A673 cells were treated with ARA-C or vehicle in triplicate and expression profiled at 24 h, 3 d, and 5 d. ARA-C regulated the expression of the EWS/FLI gene set at days 3–5 (*p* = 0.04) but not at 24 h of treatment at the EC_50_ (dose at which viability is 50% of vehicle-treated cells) (Figures 2C and S3A–S3D).

To exclude the possibility that ARA-C's modulation of the EWS/FLI signature was simply a nonspecific response to treatment with all cytotoxic agents, we asked whether other compounds known to kill Ewing sarcoma cells would induce the EWS/FLI *off* genome-wide expression pattern. Experiments were performed in triplicate. Puromycin treatment at its EC_50_ did not (Figures 2C and S3E–S3H). Thus, inhibition of cell viability alone does not induce the whole-genome effects of EWS/FLI modulation, suggesting that ARA-C has a more specific effect on EWS/FLI attenuation. As with ARA-C treatment, doxorubicin, an agent currently used to treat patients with Ewing sarcoma, did recapitulate the EWS/FLI *off* signature (*p* = 0.01) (Figures 2C and S3I–S3L) by three days. Doxorubicin was not in the National Institute of Neurological Disorders and Stroke small-molecule library.

### ARA-C Decreases EWS/FLI Protein Levels

**Figure 3:**
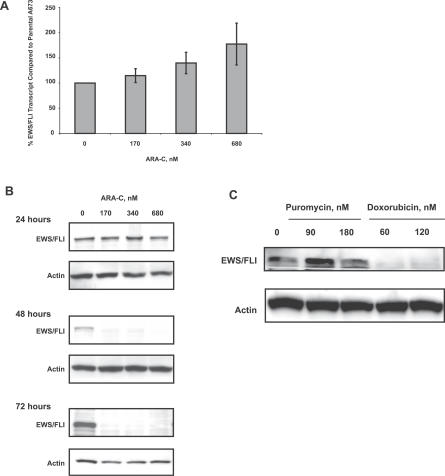
ARA-C Decreases EWS/FLI Protein Levels But Not Transcript (A) Real-time reverse transcriptase-PCR reveals increase in EWS/FLI transcript. EWS/FLI transcript was detected after ARA-C at the 3-d EC_50_ (170 nM) and 2-fold and 4-fold above. EWS/FLI expression is depicted relative to vehicle treated parental A673 cells. Error bars show the standard deviation across three replicates. (B) Western blot analysis with anti-FLI antibody confirms a marked reduction in protein levels with treatment of the A673 Ewing sarcoma cell line with ARA-C at 48 and 72 h at the 3-d ARA-C EC_50_ (170 nM), and 2-fold and 4-fold above (340 and 680 nM, respectively). Actin is shown as a loading control. (C) Western blot analysis with anti-FLI antibody confirms no reduction in EWS/FLI protein with 3 d of puromycin treatment at the 3-d EC_50_ (90 nM) or 2-fold above. With doxorubicin, there is a reduction in EWS/FLI protein levels with 3-d treatment at the 3-d EC_50_ (60 nM) and 2-fold above (120 nM). Actin is shown as a loading control.

Because the GE-HTS approach to screening does not assume prior knowledge of the target of active small molecules, ARA-C's modulation of EWS/FLI activity could occur via many possible mechanisms. For example, it might attenuate EWS/FLI by decreasing EWS/FLI transcript or protein, by interfering with EWS/FLI binding to DNA, or by interfering with downstream effectors of EWS/FLI. Quantitative PCR for EWS/FLI transcript abundance indicated no decrease in EWS/FLI mRNA levels with ARA-C treatment, a finding similar to that of the original screen. Rather, a dose-responsive increase in EWS/FLI transcript was observed (Figure 3A). In contrast, ARA-C treatment at the EC_50_ resulted in a marked decrease in EWS/FLI protein abundance by 48 h as measured by Western blotting (Figure 3B). Importantly, abundance of housekeeping genes, such as *Actin* and *GAPD,* and nuclear proteins, such as the transcription factors Sp3, YY1, and BTF3 did not decrease with ARA-C treatment (Figure S4). As observed with the analysis of genome-wide expression effects, treatment of A673 cells with puromycin at equipotent dosing did not decrease EWS/FLI protein levels, suggesting that loss of EWS/FLI is not an inevitable consequence of inhibiting A673 viability (Figure 3C). Interestingly, doxorubicin did diminish EWS/FLI protein levels, likely explaining its modulation of the *EWS/FLI* gene expression signature (Figure 3C). Similarly, the mTOR inhibitor rapamycin, previously reported to decrease EWS/FLI protein in Ewing sarcoma cell lines, induced the EWS/FLI *off* signature as assessed by our GE-HTS assay [27,28].

### ARA-C Inhibits Cell Viability in Multiple Ewing Sarcoma Cell Lines

**Figure 4:**
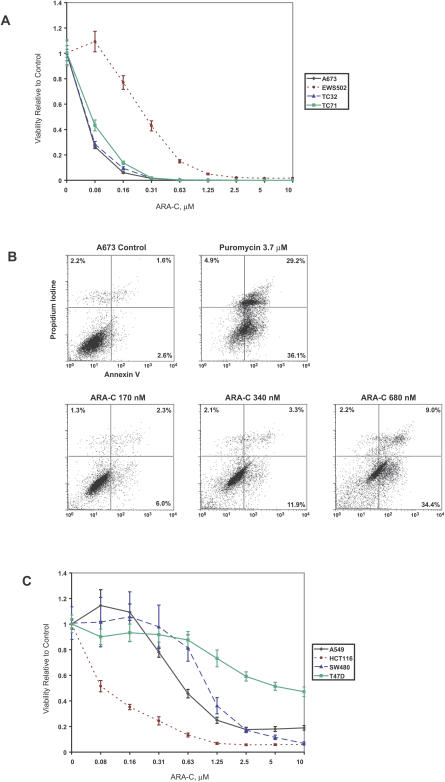
ARA-C Inhibits Cell Viability and Induces Cell Death (A) Four different Ewing sarcoma cell lines expressing the *EWS/FLI* translocation were treated with ARA-C in a dose-response series for 6 d. Cell viability was evaluated with an ATP-based assay and plotted relative to control cells. Error bars show standard deviation across four replicates. (B) A673 cells were treated with vehicle (negative control) or puromycin (positive control) or ARA-C at 170, 340, and 680 nM for 3 d, stained with annexin V-FITC and propidium iodine, and evaluated by flow cytometry. Increasing doses of ARA-C induced increased annexin V-positive cells consistent with apoptosis. (C) We treated four different carcinoma cell lines with ARA-C in a dose–response series for 6 d. Cell viability was evaluated with an ATP-based assay and plotted relative to control cells. Error bars show standard deviation across four replicates.

We evaluated the effects of ARA-C on cell viability in A673 cells and in three other Ewing sarcoma cell lines known to contain the *EWS/FLI* translocation (EWS502, TC32, and TC71) with an ATP-based assay (Figure 4A; Table S5). The EC_50_ ranged from 80 nM to 380 nM at 3 d (mean = 180 nM) and 50 nM to 280 nM at 6 d (mean = 110 nM), levels easily achievable in humans in vivo, and consistent with prior reports [29]. In fact, peak serum levels of 40 μM are routinely attainable with ARA-C treatment of patients with AML [30]. Furthermore, treatment with ARA-C induced an annexin V-positive FACS profile, consistent with apoptosis (Figure 4B).

Ewing sarcoma cell lines were found to be particularly sensitive to ARA-C, compared to non-Ewing cell lines, in which the 3 d ARA-C EC_50_ was in most cases significantly higher: colorectal adenocarcinoma SW480 (15.1 μM), human mammary adenocarcinoma T47D (40.8 μM), lung adenocarcinoma A549 (530 nM), and colorectal adenocarcinoma HCT116 (250 nM). These data suggest that not all malignant cell lines respond equally well to ARA-C treatment (Figure 4C; Table S5).

### ARA-C Abrogates Anchorage-Independent Growth

**Figure 5:**
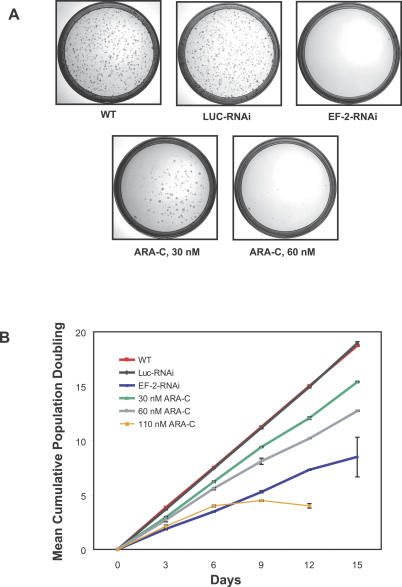
ARA-C Abrogates Anchorage-Independent Growth, a Hallmark of Oncogenic Transformation (A) ARA-C at 30 nM attenuates anchorage-independent growth as assessed by a soft agar assay and abrogates it at 60 nM as does shRNA against the EWS/FLI translocation. (B) ARA-C at 30 and 60 nM dosing decreases mean cumulative population doubling time by only 18% and 32%, respectively, at day 15. Error bars show the standard deviation across three replicates.

It had previously been reported that EWS/FLI RNAi knockdown in A673 cells abrogates anchorage-independent growth, one of the hallmarks of oncogenic transformation [14]. We sought to determine whether ARA-C would phenocopy this effect in A673 cells. We treated A673 cells with ARA-C at doses below the 3 d EC_50_ (30 and 60 nM) and evaluated colony formation in soft agar. At 30-nM ARA-C, cells were limited in their ability to form colonies. A total of 60 nM ARA-C nearly completely abrogated colony formation (Figure 5A). It is possible that loss of colony formation is merely a result of ARA-C inhibition of A673 cell viability rather than independent inhibition of transformation. In order to address this issue, we simultaneously performed a 3T10 serial passage growth assay to evaluate effects of these conditions on A673 doubling time (Figure 5B). A total of 15 d of treatment with ARA-C at 30 and 60 nM decreased mean population doubling time by 18% and 32%, respectively, compared to parental controls. Thus, while we see only modest effects of low dose ARA-C on cell growth, we see dramatic effects on transformation.

### ARA-C Inhibits Tumor Growth in Xenograft Model of Ewing Sarcoma

**Figure 6:**
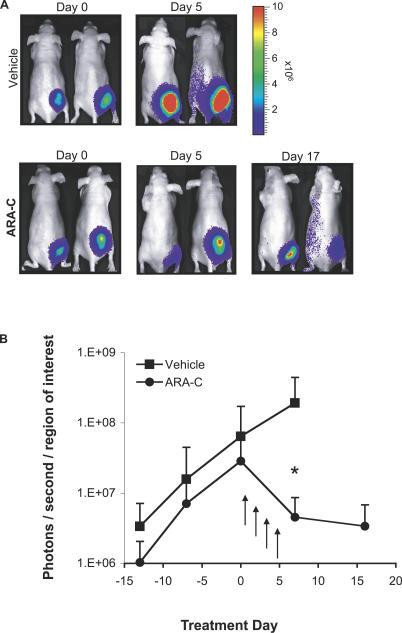
In vivo Efficacy of ARA-C in a Mouse Model of Ewing Sarcoma (A) A673-Luciferase-positive xenografts were established for 2–3 wk in NCr nude mice, with tumor burden monitored by bioluminescence imaging. Mice with logarithmically growing tumors were treated with intraperitoneal injection of ARA-C at 50 mg/kg daily for four doses or vehicle control. In both a pilot experiment A: and a larger study with cohorts of nine mice in each group B: control mice continued to have logarithmic tumor growth, necessitating sacrifice within 1 wk of starting treatments. By comparison, ARA-C-treated mice had stable disease or tumor regression with seven of nine animals showing regression. (B) Arrows indicate days of drug dosing, and the asterisk indicates *p* < 0.05 by Student's t-test (two-tailed assuming samples with equal variance). Error bars show the standard deviation across nine replicates. Bioluminescence was measured as photonic flux through standardized regions of interest (photons per second per region of interest), which did not include reflected light along the left flank (e.g., right animal in part [A] at day 17 of ARA-C treatment).

A xenograft model of Ewing sarcoma was used to evaluate the effects of ARA-C on in vivo tumor growth. A673 cells were engineered to express firefly luciferase, and tumor burden was monitored by in vivo bioluminescence imaging as previously described [14]. After inoculation of cells in the flanks of nude mice, tumors were allowed to establish for 2–3 wk. Mice with logarithmically growing tumors were divided into cohorts and treated with either systemic ARA-C at 50 mg/kg daily for four doses or vehicle control. Whereas vehicle-treated animals had tumors that continued to grow logarithmically, ARA-C-treated animals had significantly reduced tumor growth or tumor regression (Figure 6A and 6B). There was no significant effect on body weight in ARA-C treated animals, suggesting no significant systemic toxicity at this dose (unpublished data).

## Discussion

Little has changed in the treatment of patients with Ewing sarcoma over the past ten years, particularly for those with metastatic or relapsed disease. Furthermore, no current therapies target the known molecular lesion present in the vast majority of Ewing tumors, the EWS/FLI oncoprotein. One recent advance for patients with localized disease, the addition of ifosfamide and etoposide to the backbone of Ewing therapy (doxorubicin, vincristine, and cyclophosphamide), suggests that existing drugs might improve cure rates for patients with Ewing sarcoma [15]. However, a challenge has been the identification and prioritization of candidate therapies among the over 1,300 known FDA-approved drugs. A second challenge has been the difficulty in identifying, with traditional small-molecule library screening, modulators of oncoproteins involving transcription factors such as EWS/FLI. The work presented here addresses these two challenges. A gene expression–based approach was used to screen a small-molecule library, enriched for FDA-approved drugs (~750 of the 1,300 FDA-approved drugs), for compounds that attenuate EWS/FLI. ARA-C was identified as the top hit.

One of the powers of the GE-HTS approach lies in the use of multigene signatures to represent a complex biological state change. The individual signature genes are not required to be immediate transcriptional targets of the oncoprotein in question, but rather genes that are faithfully dysregulated by it. As such, the EWS/FLI *off* signature genes may be direct or indirectly regulated by EWS/FLI. Previously reported, putative direct EWS/FLI target genes are not represented in our EWS/FLI *off* signature, possibly in part because the signature genes are not direct targets of EWS/FLI. However, this difference likely also reflects a difference in the cell system used in our screen. Because the true cell of origin for Ewing sarcoma is unknown, many of the previously reported EWS/FLI target genes were identified in heterologous cell systems, such as NIH3T3 cells. As noted in Smith et al. [14], a discrepancy can be seen between identified EWS/FLI targets in heterologous systems versus those identified in Ewing sarcoma, itself. For example, Smith et al. found that *MYC, ID2, MFNG, KRT115, UBE2C, CYP2FI,* and *CDKN1C* were not significantly altered as previously reported in the literature based upon heterologous systems [14]. The use of human Ewing sarcoma cell lines rather than heterologous systems to define the signature of EWS/FLI *off* should more closely recapitulate the activity of EWS/FLI in human disease.

ARA-C was the top hit confirmed to induce both the EWS/FLI *off* signature and broader gene expression changes associated with the EWS/FLI o*ff* state. These expression changes are secondary to ARA-C induced decrease in EWS/FLI protein. As with EWS/FLI knockdown, ARA-C treatment inhibits transformation and cell viability. Thus, it seems likely that the biological effects of ARA-C are, at least in part, related to the decrease in EWS/FLI protein. However, ARA-C has a more profound effect on A673 cell growth and apoptosis than does EWS/FLI knockdown via RNAi. This difference in the effects of RNAi directed against EWS/FLI versus ARA-C treatment in A673 may be explained by the inability of the shRNAs to completely ablate EWS/FLI expression. Indeed, we note that it is difficult to maintain long-term knockdown of EWS/FLI, suggesting that there is strong negative selection for EWS/FLI-ablated cells. We also note that Prieur et al. [12] have reported that two rounds of siRNA-mediated knockdown of EWS/FLI does in fact induce apoptosis. Lastly, it is certainly possible (if not likely) that ARA-C has additional cytotoxic effects independent of its effect on EWS/FLI expression. ARA-C is a known nucleoside analogue, which is transported inside cells and then phosphorylated to the triphosphate form by deoxycytidine kinase. The incorporation of phosphorylated ARA-C into DNA inhibits further DNA synthesis and causes death by apoptosis [30].

ARA-C is not generally considered an agent that interferes with protein synthesis or enhances degradation. We have shown with real-time PCR that ARA-C is not decreasing transcript as the mechanism of the decrease in EWS/FLI protein. A second possibility is that ARA-C inhibits protein synthesis by a mechanism not well characterized. A third possibility is that ARA-C increases the degradation of EWS/FLI. Interestingly, doxorubicin, a chemotherapy agent that is the backbone of current Ewing sarcoma therapy, also decreased EWS/FLI protein and induced the EWS/FLI *off* gene expression signature, and rapamycin, an mTOR inhibitor previously reported to decrease EWS/FLI protein, induces an EWS/FLI *off* signature. Whether this is a universal phenomenon shared in common by clinically effective drugs for Ewing sarcoma is yet to be determined. Certainly it will be of interest to establish the mechanism by which ARA-C regulates EWS/FLI protein abundance, but given ARA-C's well-characterized safety profile, these findings are of immediate clinical relevance for a disease where relapse is nearly always incurable.

The EC_50_ level of ARA-C (50–280 nM) is well below clinically achievable serum levels in humans. With high dose ARA-C (3 g/m^2^/dose every 12 h), serum levels up to 40 μM ARA-C are obtained, at 2 g/m^2^/day, 5-μM levels are obtained, and at 200 mg/m^2^/day, 100–500-nM levels are obtained [30]. Currently, there is no curative therapy for patients with recurrent Ewing sarcoma, and much room remains for improvement in the treatment of patients with newly diagnosed disease, particularly those with metastases. There is a long experience of treating patients, both adults and children, with AML and acute lymphoblastic leukemia with ARA-C, with established dosing regimens and well-characterized toxicity profiles [31–33]. We therefore believe that clinical trials testing ARA-C in patients with Ewing sarcoma are warranted.

We have demonstrated that development of expression-based small-molecule library screening enables identification of modulators of important oncoproteins previously considered undruggable. Furthermore, it identified an already FDA-approved candidate that can now be brought to clinical trial for children with Ewing sarcoma. A national clinical trial to test ARA-C in patients with relapsed Ewing sarcoma is currently in development. In principle, this approach can be utilized to identify modulators of any oncoprotein, simply by defining a signature of the oncoprotein's activity. With an ever-expanding list of “undruggable” oncoproteins critical in the development of cancer, such as the discovery of ETS transcription factor rearrangements in the majority of patients with prostate cancer [34,35], it will be increasingly important to explore alternative small-molecule discovery platforms. GE-HTS provides an avenue for identifying tool compounds for the study of these genetic lesions.

## Supporting Information

Figure S1 ARA-C Induces an EWS/FLI *Off* Signature in A673 Ewing Cells (15 KB PDF)

Figure S2 ARA-C Does Not Induce a “Pseudo” EWS/FLI *Off* Signature in AML (23 KB PDF)

Figure S3 GSEA Results for ARA-C, Puromycin, and Doxorubicin (142 KB PDF)

Figure S4 ARA-C Does Not Decrease Nuclear Protein Levels (91 KB PDF)

Table S1 LMA Probe Sequence (16 KB PDF)

Table S2 EWS/FLI Modulation Gene Sets for GSEA (45 KB PDF)

Table S3 GSEA Results for EWS/FLI Modulation Signature (10 KB PDF)

Table S4 Affymetrix U133A Ewing Sample Names (28 KB PDF)

Table S5 Cell Line Viability Data (18 KB PDF)

## Abbreviations

AML: acute myeloblastic leukemia
ARA-C: cytosine arabinoside
GAPD: Glyceraldehyde-3-phosphate dehydrogenase
GE-HTS: gene expression-based high-throughput screening
GSEA: gene set enrichment analysis
LMA: ligation-mediated amplification
RNAi: RNA interference
shRNA: short hairpin RNA
