# Supplementary material for: Signature-Based Small Molecule Screening Identifies Cytosine Arabinoside as an EWS/FLI Modulator in Ewing Sarcoma
Source: PLoS Med. 2007 Apr 10;4(4):e122. doi: 10.1371/journal.pmed.0040122 (PMC1851624; doi:10.1371/journal.pmed.0040122)
Supplement: Supplementary file 1 [file pmed.0040122.sg001.pdf]

Supplementary Figure 1: ARA-C Induces an EWS/FLI *Off* Signature in A673 Ewing Sarcoma Cells

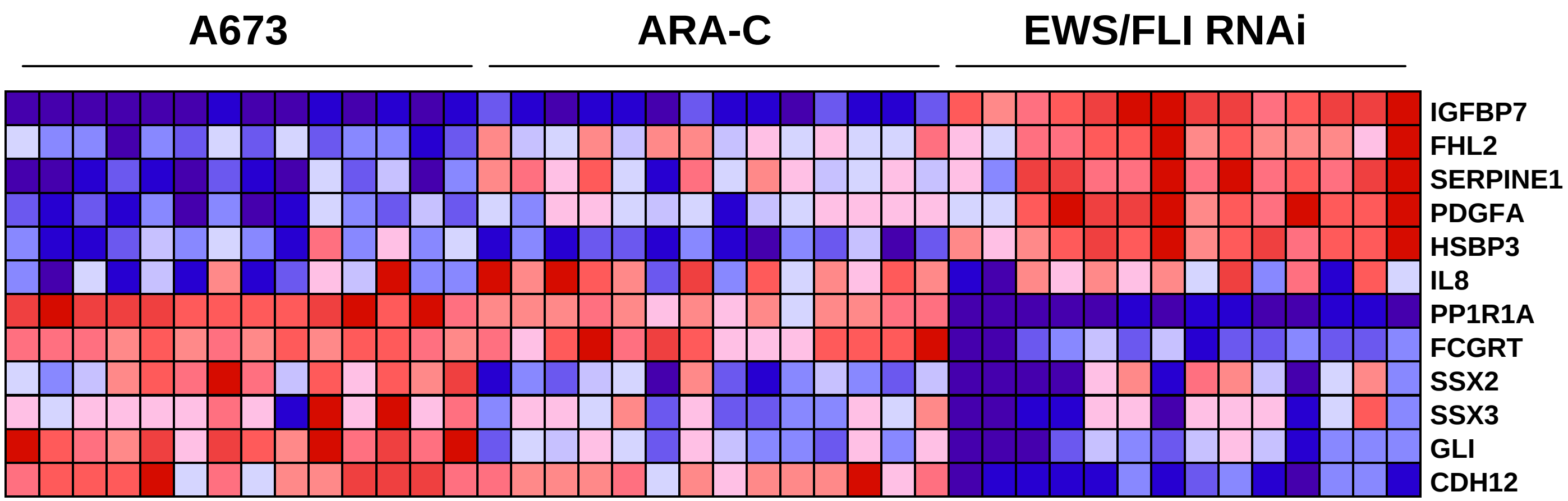

A673 cells were treated in replicate of 14 with vehicle or at the ARA-C EC50 and the EWS/FLI *off* signature genes evaluated with the GE-HTS assay. A673 with EWS/FLI directed shRNA are included as controls. *SCA1* and *SP100* were removed from the analysis secondary to poor performance in the controls. ARA-C treatment induced the appropriate direction of change in the majority of marker genes consistent with EWS/FLI *off*. Samples are shown in columns and genes in rows as a ratio to the control gene, *GAPD*. Blue represents poorly expressed genes and red depicts highly expressed genes with row normalization.
