# Supplementary material for: Signature-Based Small Molecule Screening Identifies Cytosine Arabinoside as an EWS/FLI Modulator in Ewing Sarcoma
Source: PLoS Med. 2007 Apr 10;4(4):e122. doi: 10.1371/journal.pmed.0040122 (PMC1851624; doi:10.1371/journal.pmed.0040122)
Supplement: Supplementary file 2 [file pmed.0040122.sg002.pdf]

Supplementary Figure 2A: ARA-C Does Not Induce a “Pseudo” EWS/FLI *Off* Signature in HL-60 AML cells

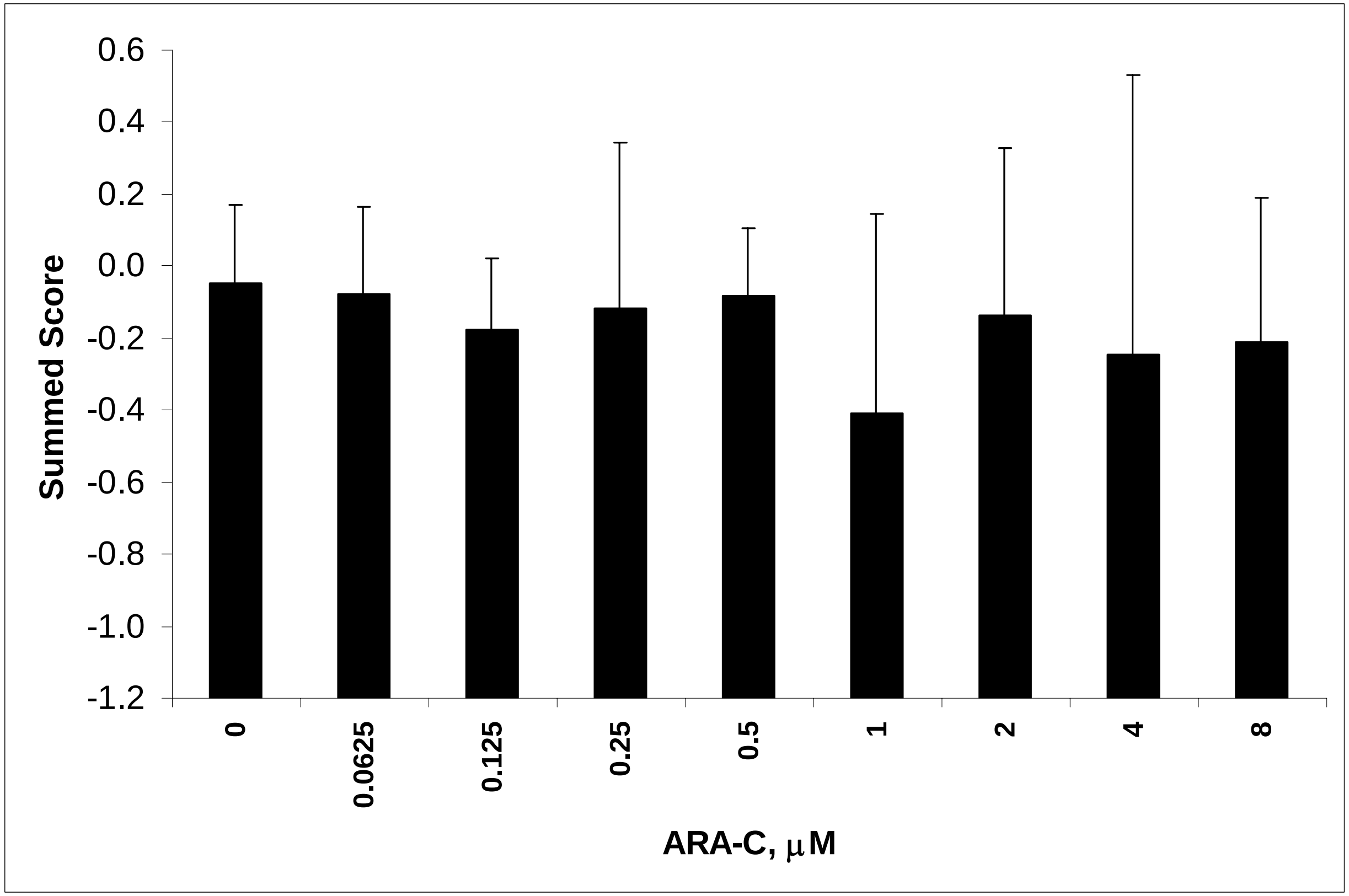

HL-60 cells were treated with ARA-C in a dose-response series designed to include the EC50 for 3 days. The EWS/FLI *off* signature (Summed Score) was evaluated with the GE-HTS assay. Error bars show the standard deviation across 14 replicates. ARA-C does not induce a “pseudo” EWS/FLI *off* signature in this AML cell line that does not express EWS/FLI.

Supplementary Figure 2B: ARA-C Does Not Induce a "Pseudo" EWS/FLI *Off* Signature in U937 AML cells

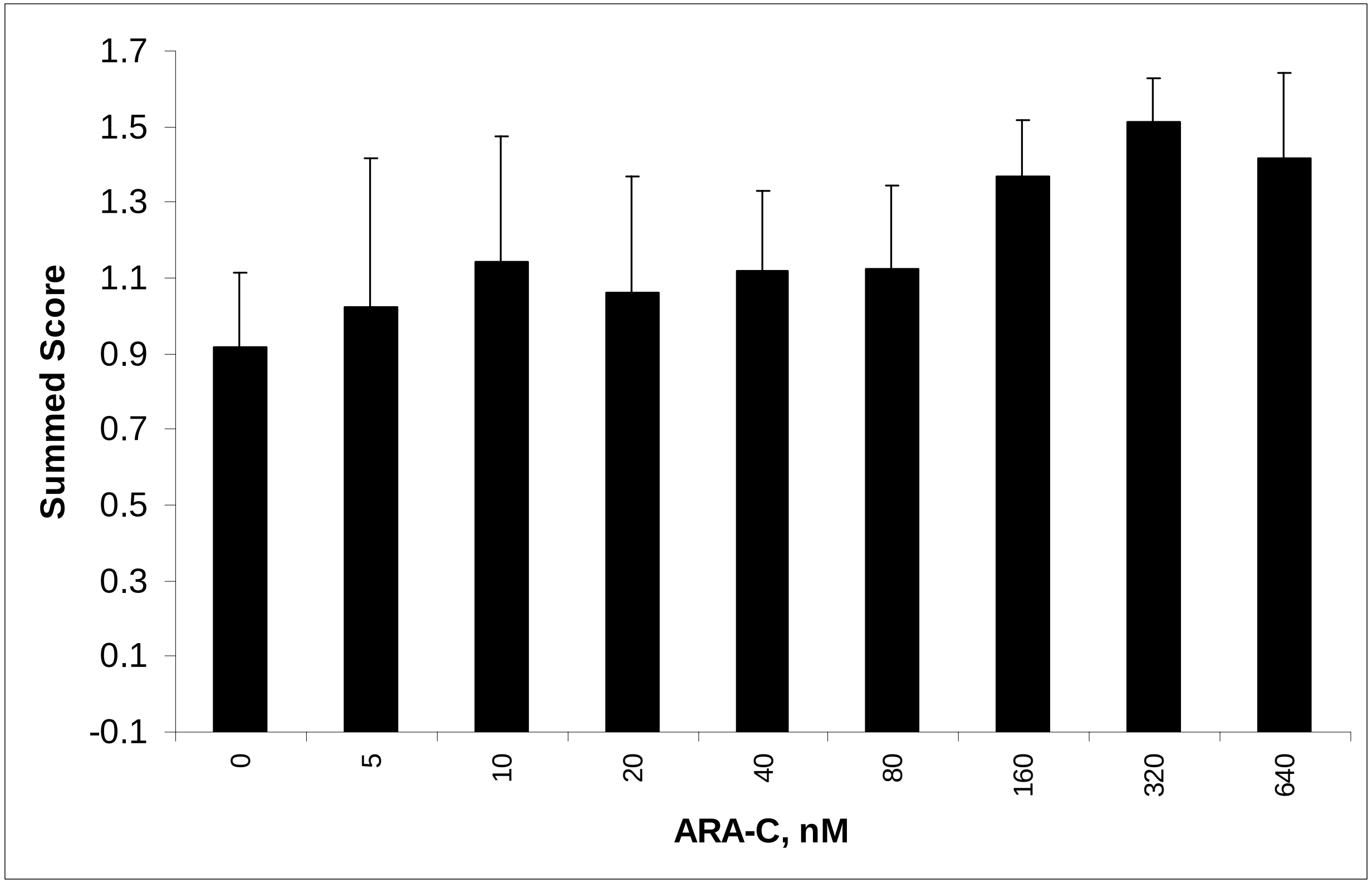

U937 cells were treated with ARA-C in a dose-response series designed to include the EC50 for 3 days. The EWS/FLI *off* signature (Summed Score) was evaluated with the GE-HTS assay. Error bars show the standard deviation across 14 replicates. ARA-C does not induce a "pseudo" EWS/FLI *off* signature in this AML cell line that does not express EWS/FLI.
