# Supplementary material for: Signature-Based Small Molecule Screening Identifies Cytosine Arabinoside as an EWS/FLI Modulator in Ewing Sarcoma
Source: PLoS Med. 2007 Apr 10;4(4):e122. doi: 10.1371/journal.pmed.0040122 (PMC1851624; doi:10.1371/journal.pmed.0040122)
Supplement: Supplementary file 3 [file pmed.0040122.sg003.pdf]

Supplementary Figures 3A-L
(GSEA Results for ARA-C, Puromycin, and Doxorubicin)

GSEA results for the set of EWS/FLI shRNA upregulated genes confirmed in the inducible EWS/FLI data set (66 genes) tested at 24 hours, 3 and 5 days at the EC50 for ARA-C (170 nM), puromycin (90 nM), and doxorubicin (60 nM) in replicate of three. The running enrichment score, generated by the markers after they have been ordered with the phenotype of interest, is shown in red. The position of the genes in the gene set is shown as a blue line ("Hits"). The ranking scores for the genes are shown in grey. The amount of enrichment is estimated by the maximum deviation from zero of the enrichment score (ES) and statistical significance was determined using permutation testing (2500 permutations).

Supplementary Figure 3A : GSEA Results for 170 nM ARA-C at 24 Hours

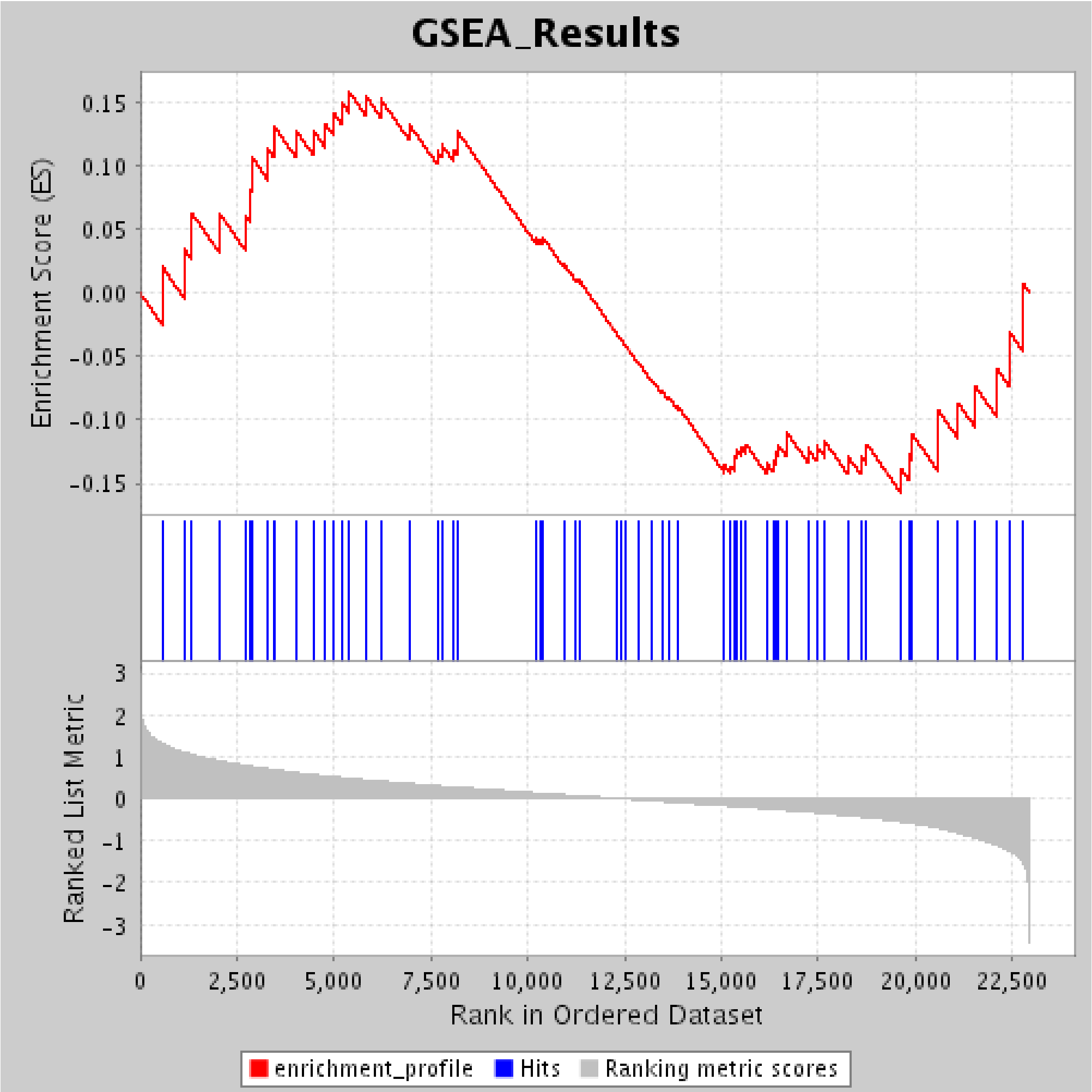

(ES=-0.16, P=0.947)

Supplementary Figure 3B: GSEA Results for 170 nM ARA-C at 3 Days

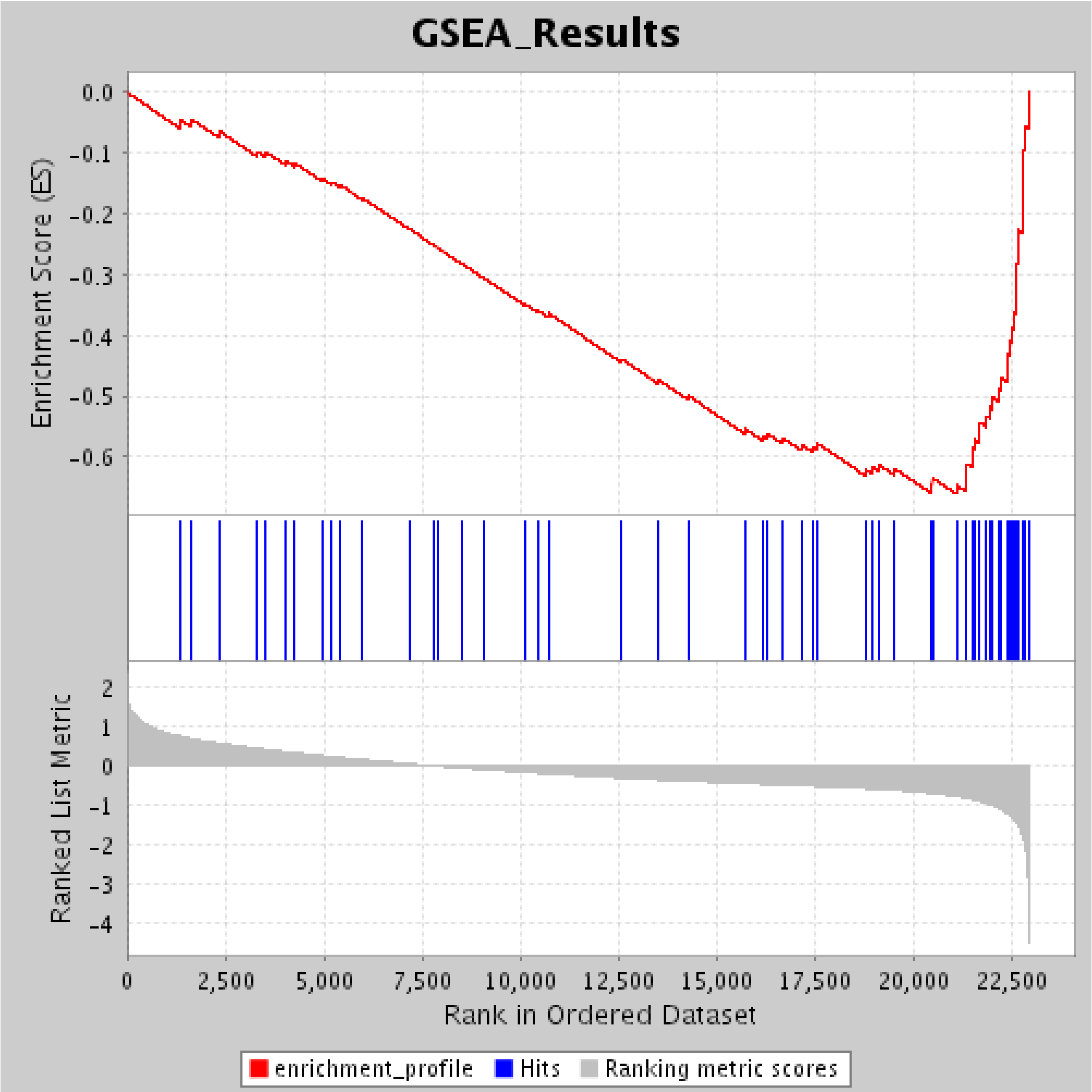

(ES=-0.66, P=0.008)

Supplementary Figure 3C: GSEA Results for 170 nM ARA-C at 5 Days

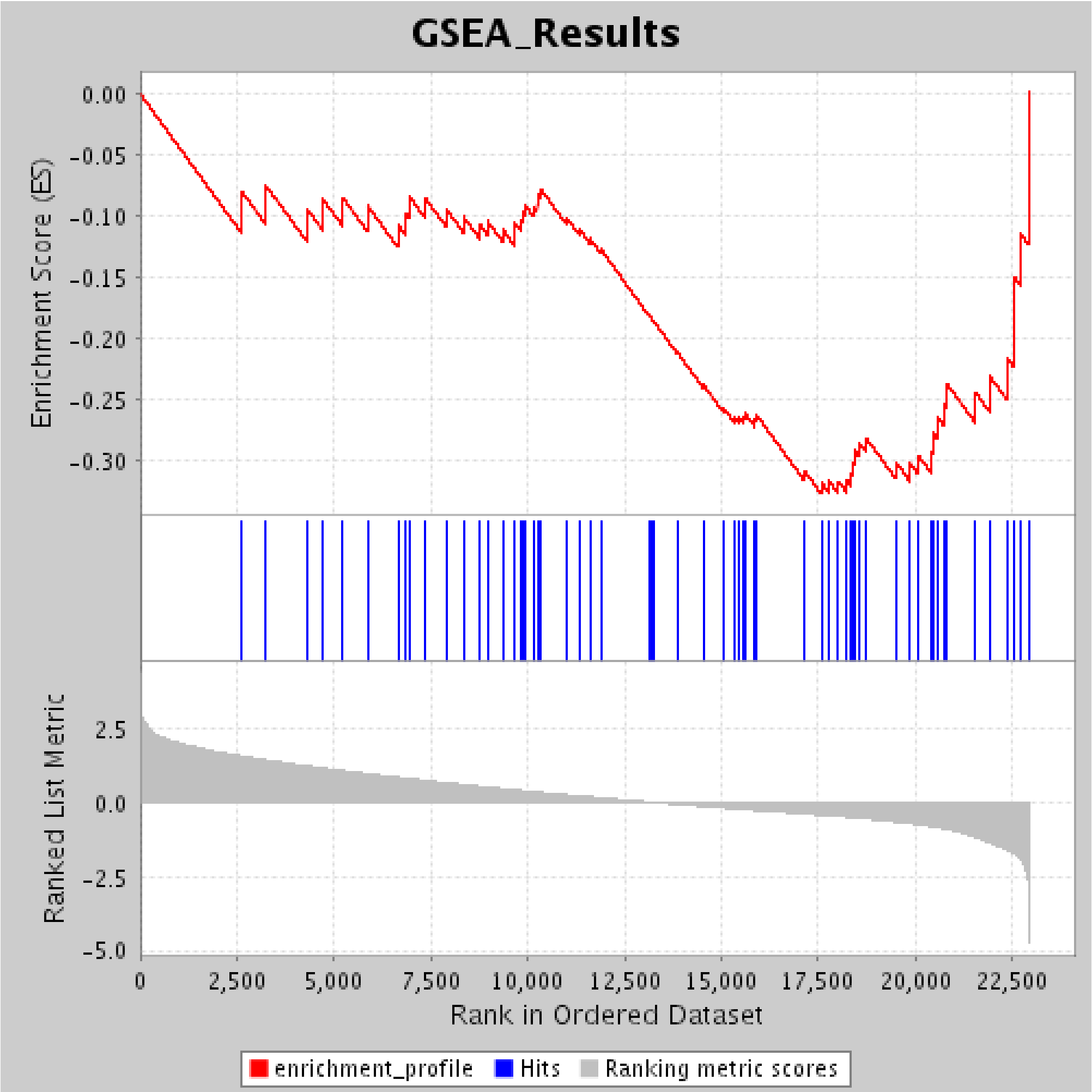

(ES=-0.33, P=0.464)

Supplementary Figure 3D: GSEA Results for 170 nM ARA-C at 3 and 5 Days Combined

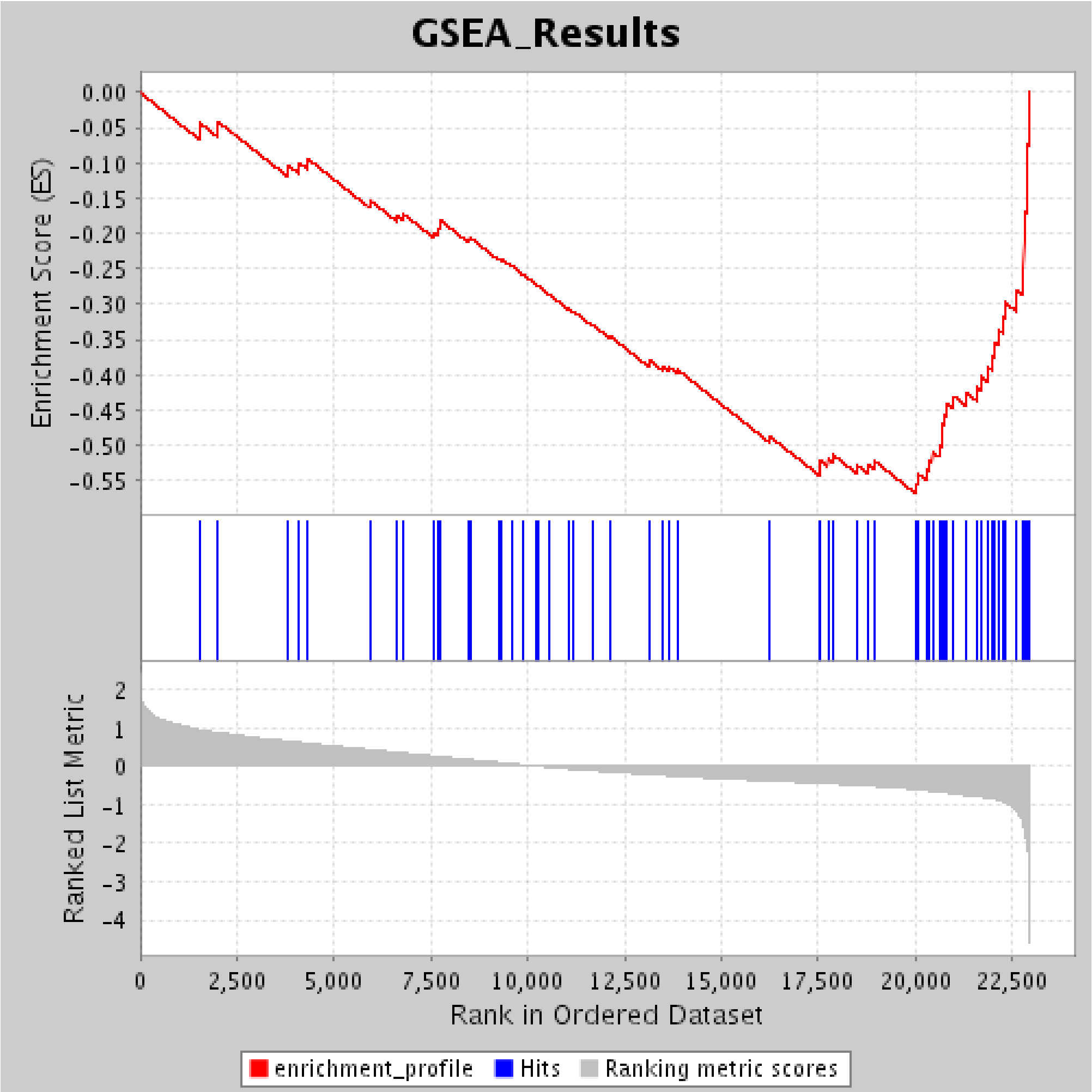

(ES=-0.57, P =0.04)

Supplementary Figure 3E: GSEA Results for 90 nM Puromycin at 24 Hours

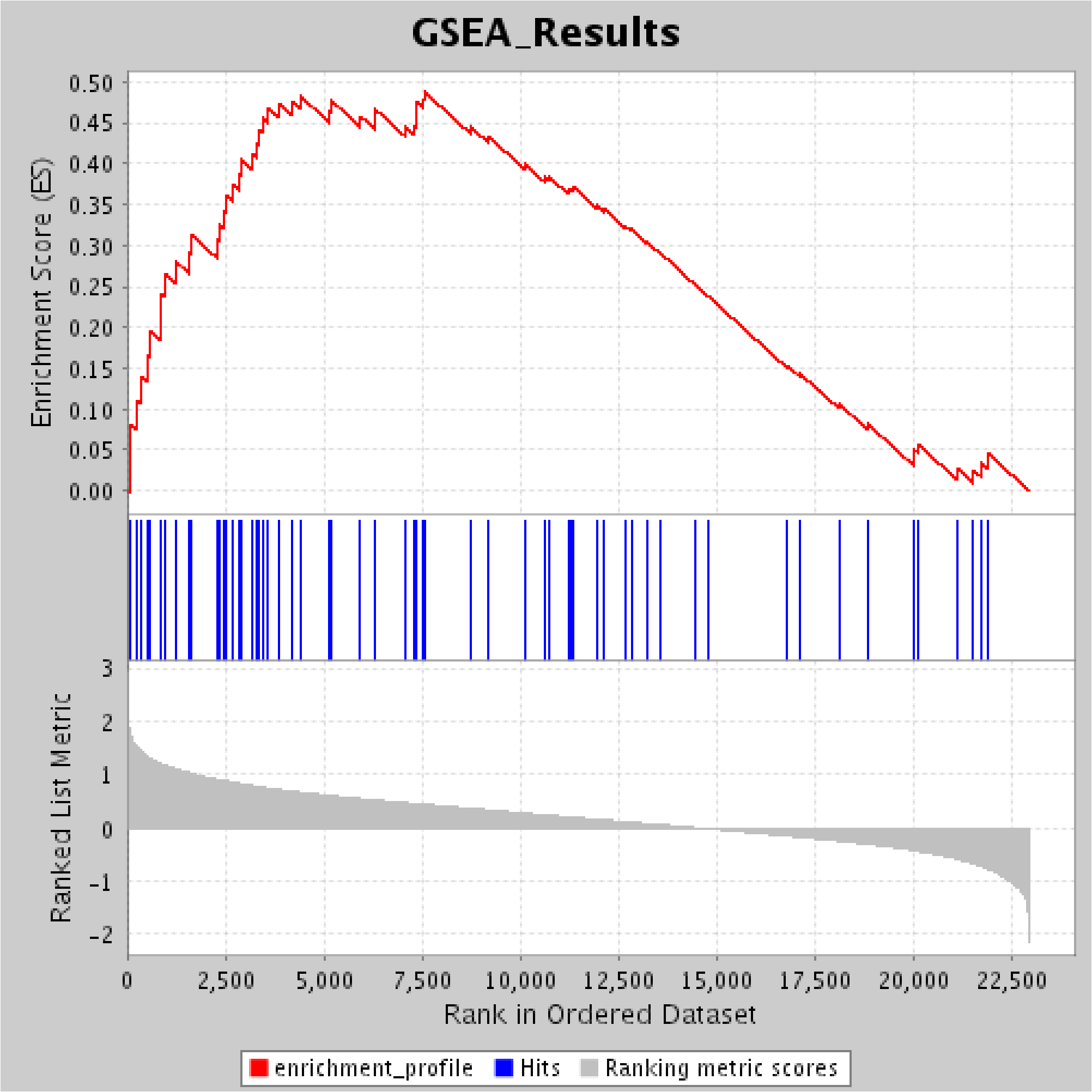

(ES=0.49, P=0.053)

Supplementary Figure 3F: GSEA Results for 90 nM Puromycin at 3 Days

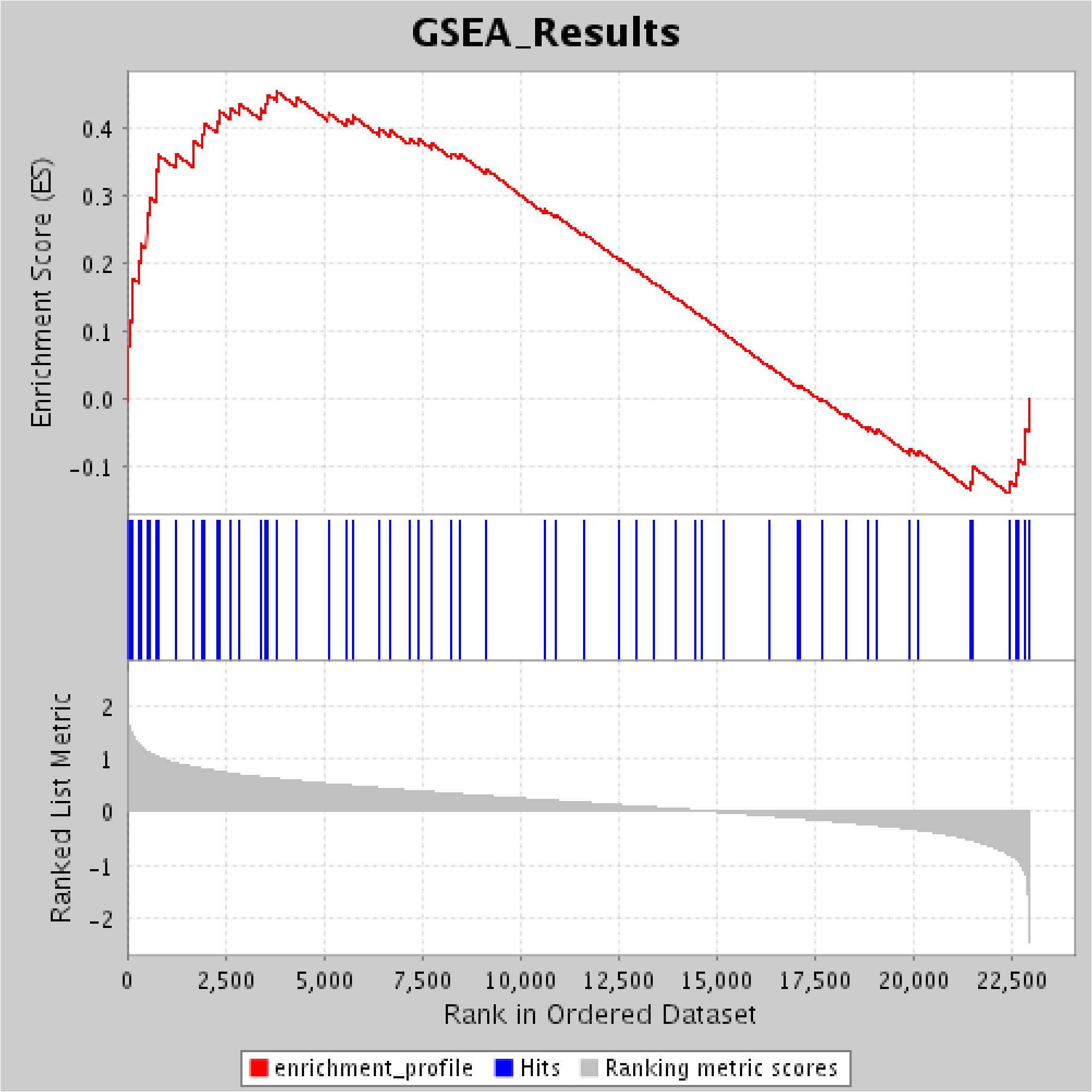

(ES=0.45, P=0.065)

Supplementary Figure 3G: GSEA Results for 90 nM Puromycin at 5 Days

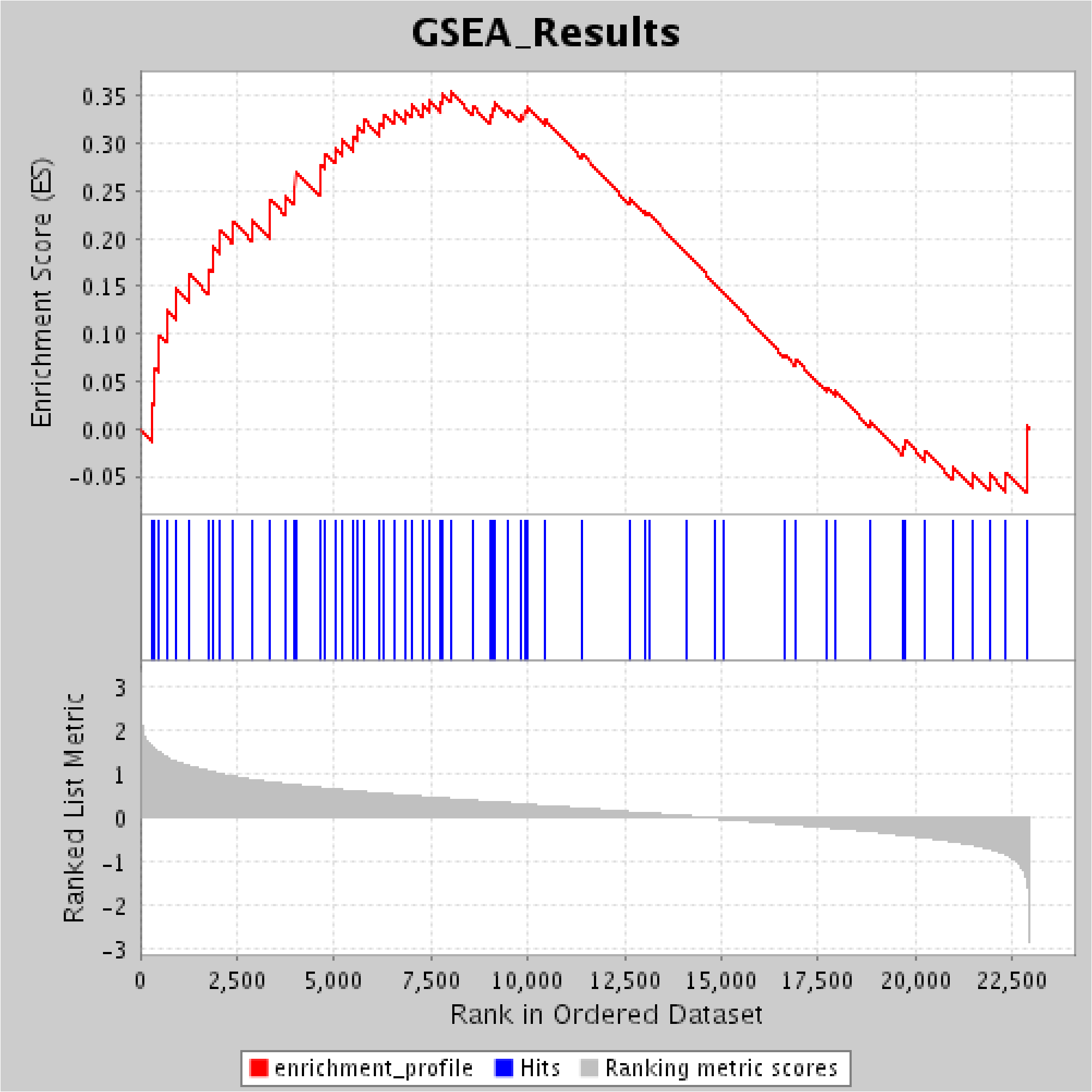

(ES=0.35, P=0.369)

Supplementary Figure 3H: GSEA Results for 90 nM Puromycin at 3 and 5 Days Combined

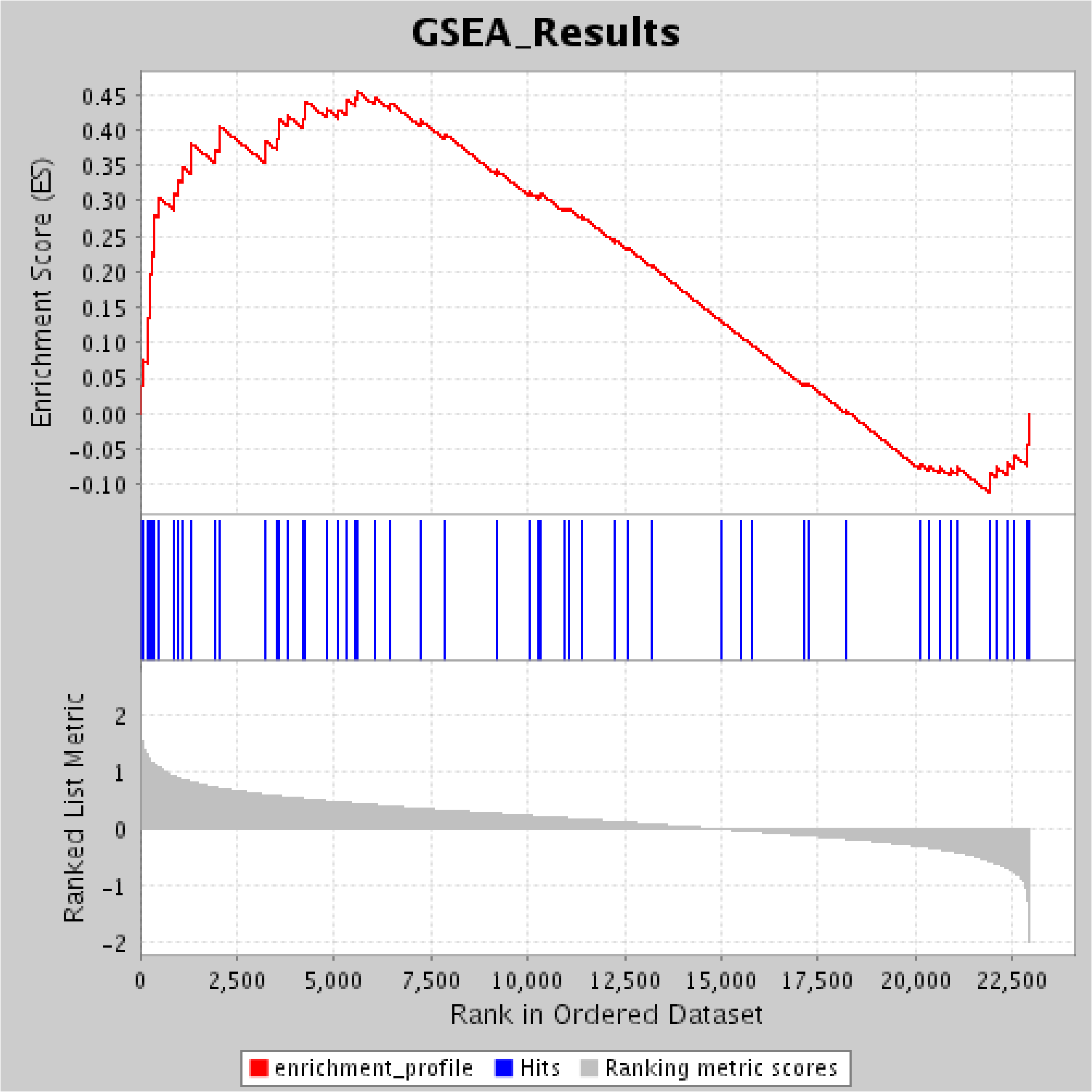

(ES=0.45, P=0.064)

Supplementary Figure 3I: GSEA Results for 60 nM Doxorubicin at 24 Hours

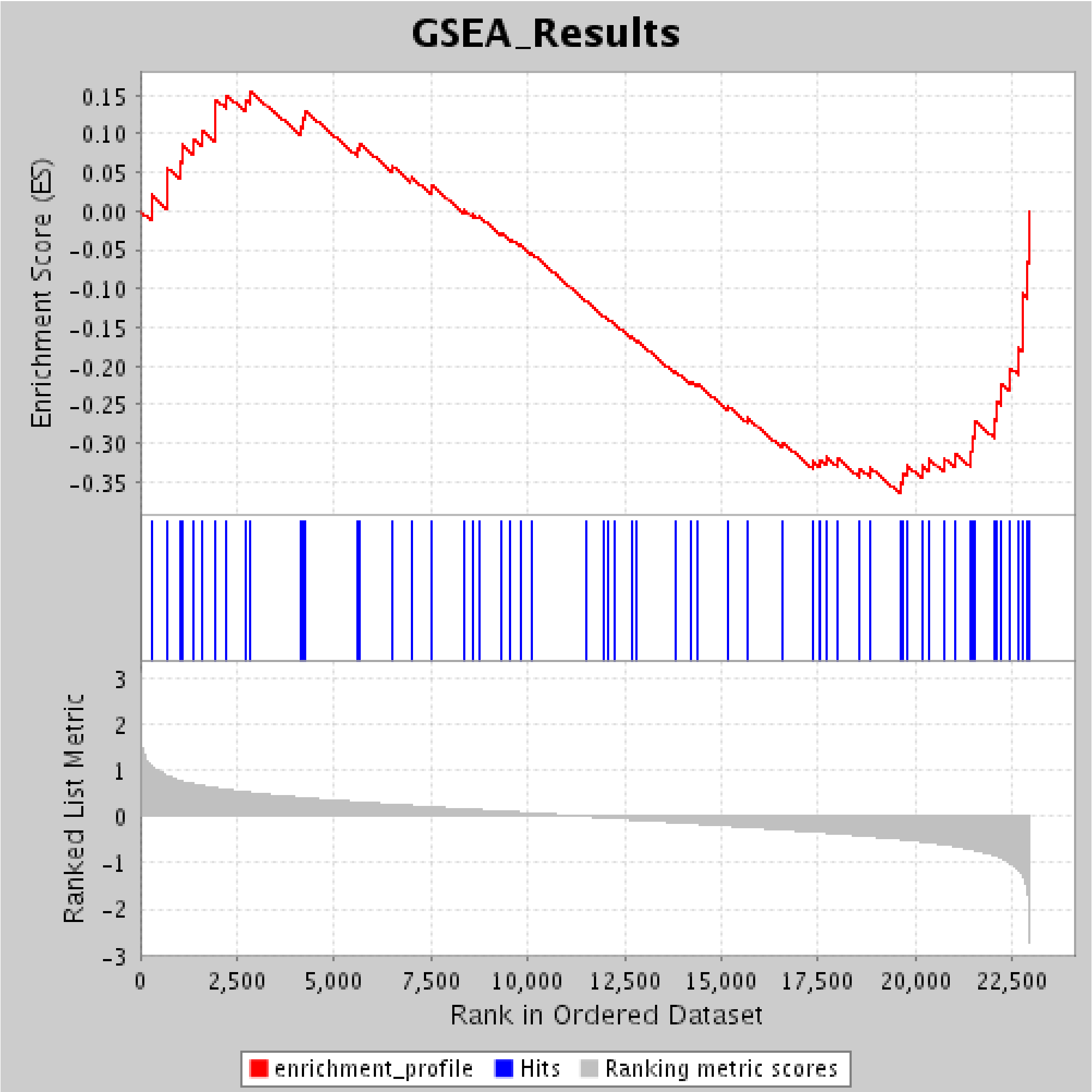

(ES=-0.37, P=0.317)

Supplementary Figure 3J: GSEA Results for 60 nM Doxorubicin at 3 Days

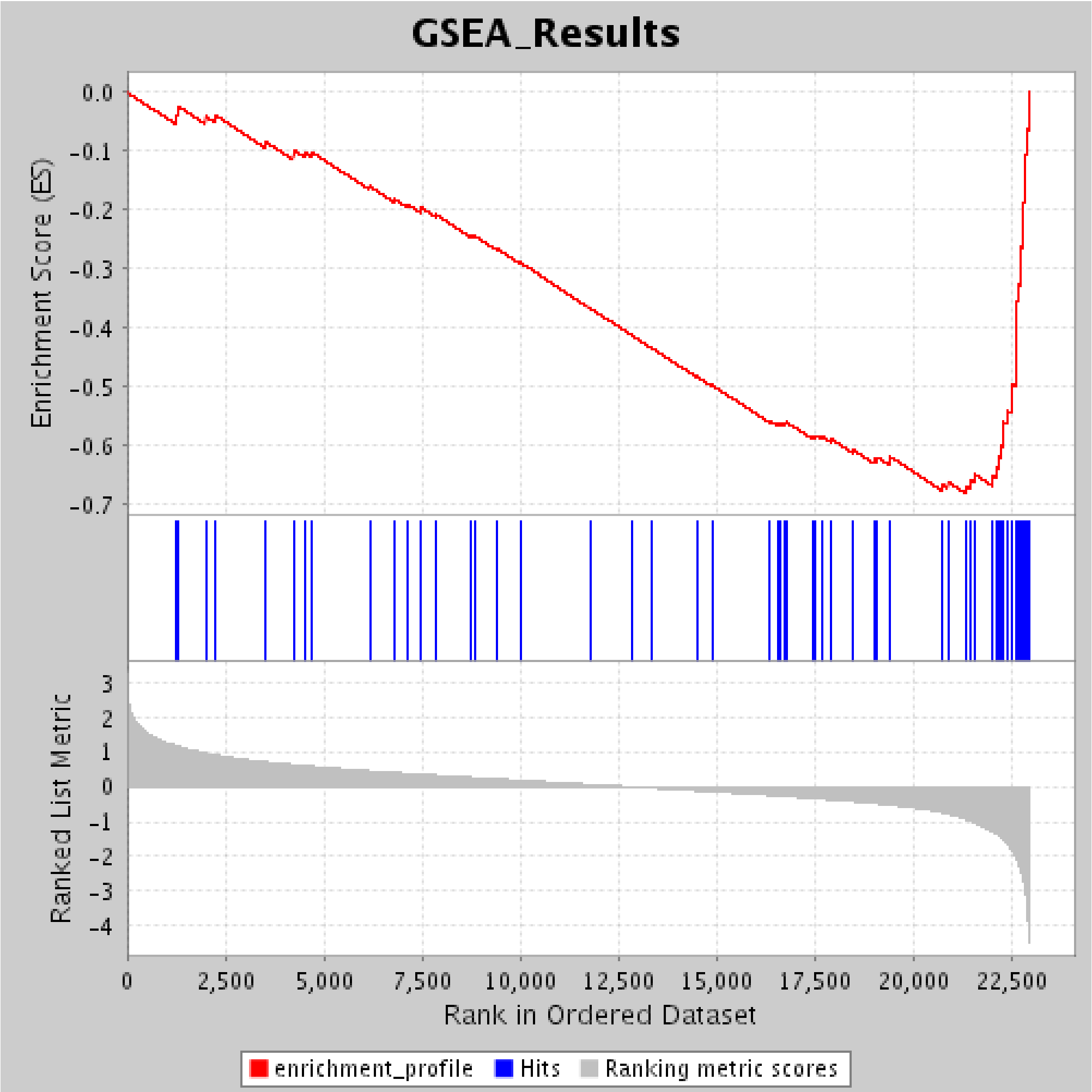

(ES=-0.68, P<0.001)

Supplementary Figure 3K: GSEA Results for 60 nM Doxorubicin at 5 Days

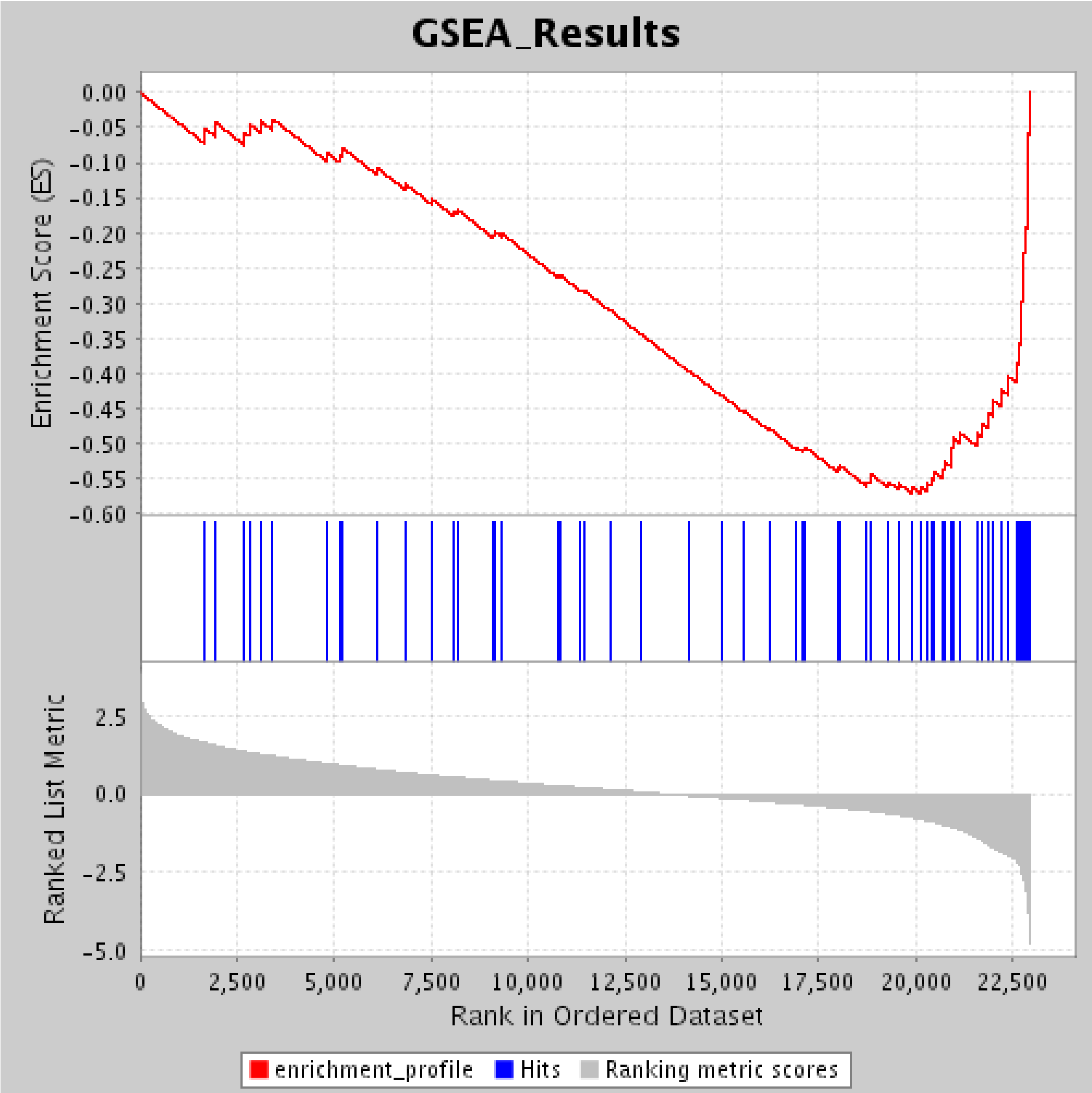

(ES=-0.57, P=0.034)

Supplementary Figure 3L: GSEA Results for 60 nM Doxorubicin at 3 and 5 Days Combined

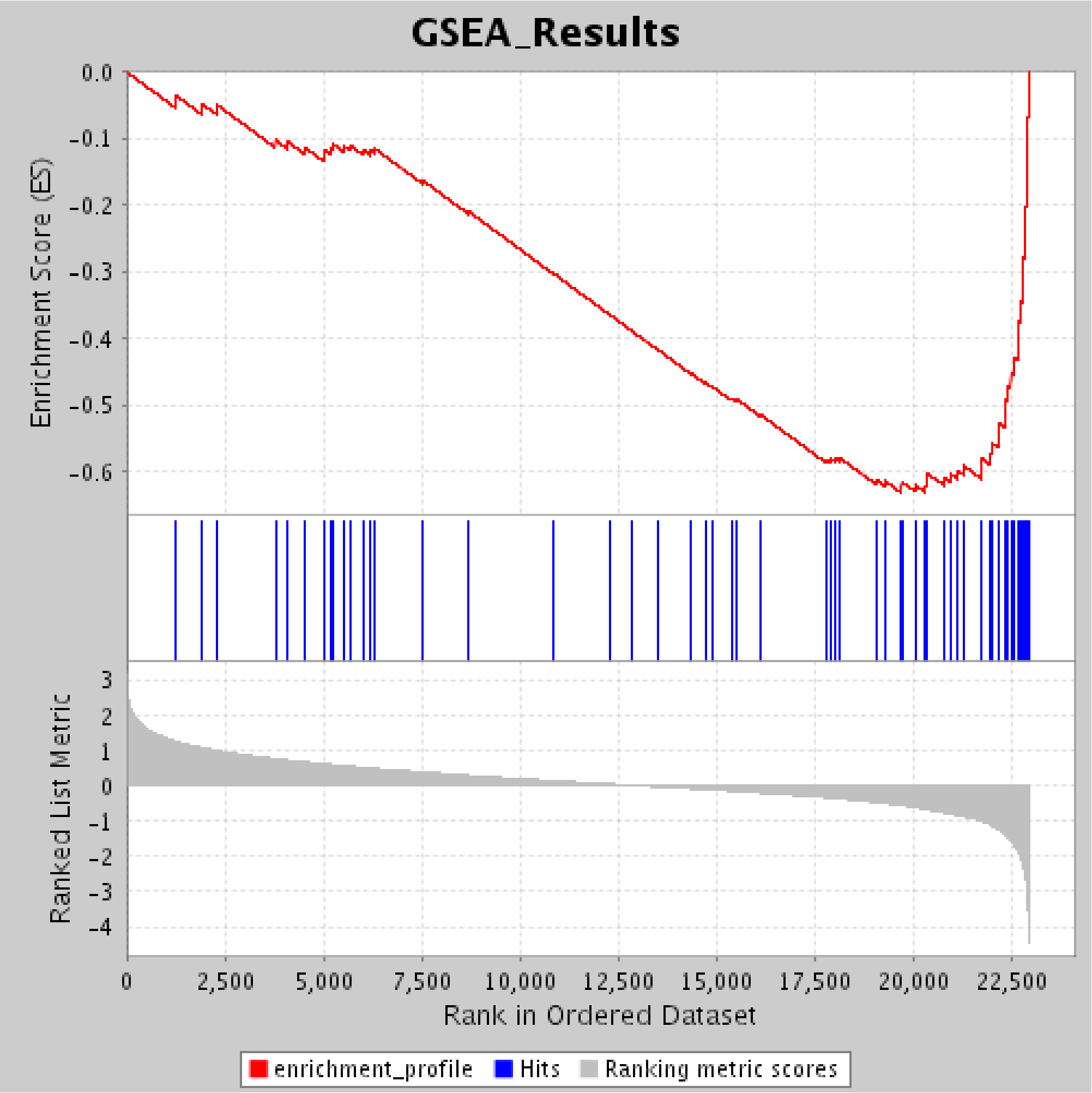

(ES=-0.63, P=0.013)
