# Supplementary material for: Signature-Based Small Molecule Screening Identifies Cytosine Arabinoside as an EWS/FLI Modulator in Ewing Sarcoma
Source: PLoS Med. 2007 Apr 10;4(4):e122. doi: 10.1371/journal.pmed.0040122 (PMC1851624; doi:10.1371/journal.pmed.0040122)
Supplement: Supplementary file 4 [file pmed.0040122.sg004.pdf]

Supplementary Figure 4: ARA-C Does Not Decrease Nuclear Protein Levels

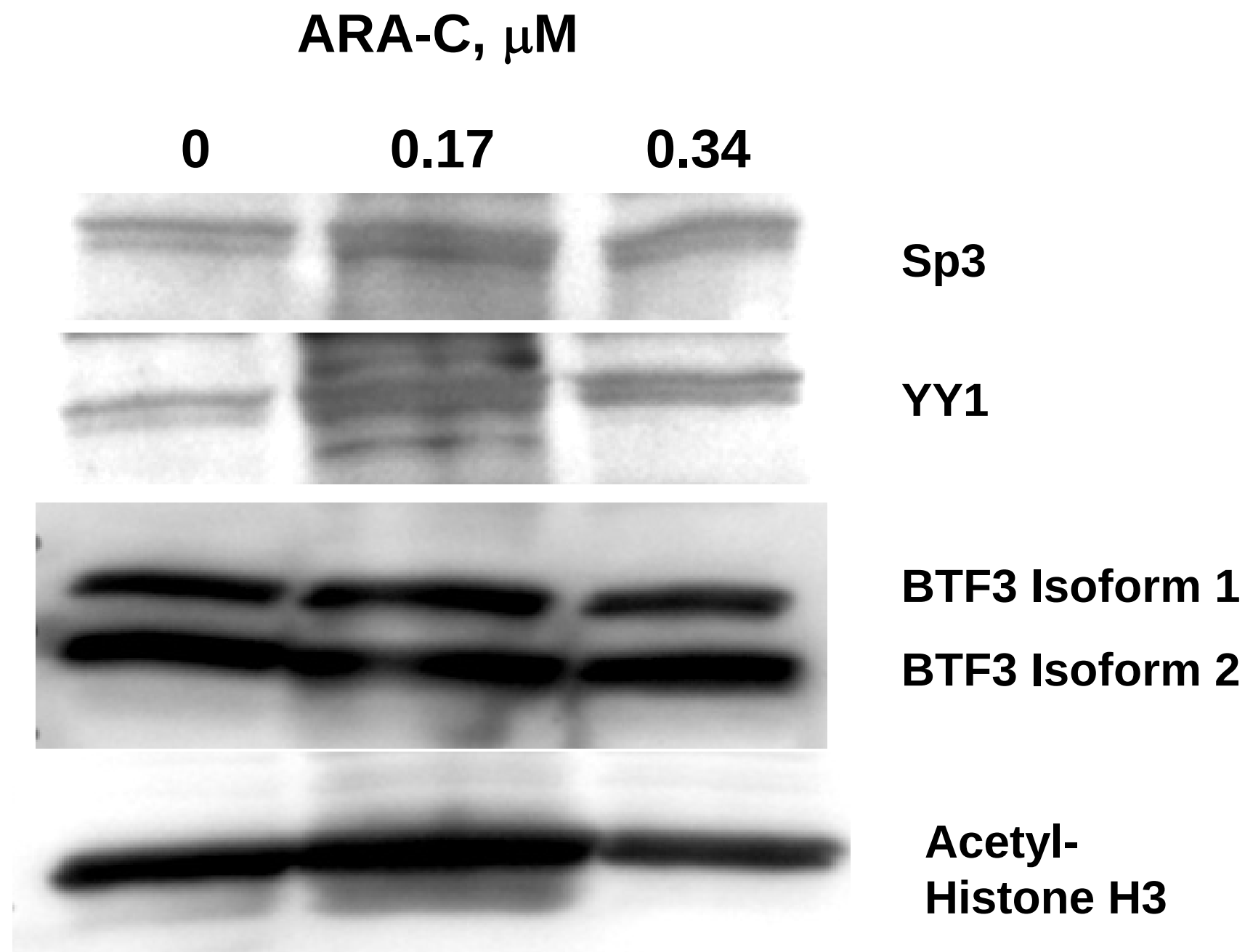

Western blot analysis with anti-Sp3, anti-YY1, anti-BTF3, and anti-acetyl-Histone H3 antibody confirms no reduction in protein levels for these three transcription factors (Sp3, YYI, and BTF3) nor for Histone H3 in nuclear extracts from A673. Cells were treated for three days with ARA-C at 0.17 or 0.34 μM and compared to control A673 cells.
