# Supplementary material for: Signature-Based Small Molecule Screening Identifies Cytosine Arabinoside as an EWS/FLI Modulator in Ewing Sarcoma
Source: PLoS Med. 2007 Apr 10;4(4):e122. doi: 10.1371/journal.pmed.0040122 (PMC1851624; doi:10.1371/journal.pmed.0040122)
Supplement: Supplementary file 5 [file pmed.0040122.st001.pdf]

Supplementary Table 1

LMA Probes (EWS/FLI Knockdown Signature Genes)

Gene Name	FlexMap Tag #	Probe Sequence
IGFBP7	33	5’-TAATACGACTCACTATAGGGTCAATTACTTCACTTTAATCCTTTCATGGTTAAAAGTAGTCATG-3’<br>5’-PO4-GATAACTACATTACCTGTTCTCCCTTTAGTGAGGGTTAAT-3’
GAPD	34	5'-TAATACGACTCACTATAGGGTCATTCATATACATACCAATTCATATCTCCCCTCCTCACAGTTG -3'<br>5'-PO4-CCATGTAGACCCCTTGAAGATCCCTTTAGTGAGGGTTAAT-3'
SCA1	35	5'-TAATACGACTCACTATAGGGCAATTTCATCATTCATTCATTTCACGTGGTGTGTCTATTTGTAG-3’<br>5'-PO4-GATACACTCGAGTCTGTTTATCCCTTTAGTGAGGGTTAAT-3'
HSBP3	36	5'-TAATACGACTCACTATAGGGCAATTCATTTCATTCACAATCAATATGACATGGGGAAGATGATG-3'<br>5'-PO4-GTTCAGCCACTGGTACTACGTCCCTTTAGTGAGGGTTAAT-3'
SP100	38	5'-TAATACGACTCACTATAGGGTCAATCATTACACTTTTCAACAATCACAAAGCCAATCTCAATCC-3'<br>5'-PO4-CTCAAAACAATCTTGTCATATCCCTTTAGTGAGGGTTAAT-3'
PP1R1A	39	5'-TAATACGACTCACTATAGGGTACACAATCTTTTCATTACATCATAGCCTGAGAAGCGTCAGGTC-3'<br>5'-PO4-CCACTTCTGCCATGCAGCTATCCCTTTAGTGAGGGTTAAT-3'
CDH12	40	5'-TAATACGACTCACTATAGGGCTTTCTACATTATTCACAACATTATTATGTCAAGAAGCAATAGC-3'<br>5'-PO4-GACACTCTGACTCTCCTCAT -TCCCTTTAGTGAGGGTTAAT-3'
IL8	41	5'-TAATACGACTCACTATAGGGTTACTACACAATATACTCATCAATTTATCAAAGAACTGAGAGTG -3'<br>5'-PO4-ATTGAGAGTGGACCACACTGTCCCTTTAGTGAGGGTTAAT-3'
SSX2	42	5'-TAATACGACTCACTATAGGGCTATCTTCATATTTCACTATAAACAGTACCAGTATAAGCATCTG-3'<br>5'-PO4-CCATCTGCTTTTCCCATTGCTCCCTTTAGTGAGGGTTAAT-3'
FCGRT	43	5'-TAATACGACTCACTATAGGGCTTTCAATTACAATACTCATTACAATCTCTGAGCCTCCAGAAGG-3'<br>5'-PO4- GGTTCTGGGCCTAGTTGTCCTCCCTTTAGTGAGGGTTAAT-3'
FHL2	44	5'-TAATACGACTCACTATAGGGTCATTTACCAATCTTTCTTTATACCTCACTGTCTATTTAAGTGC-3'<br>5'-PO4-AATTAACATATGTCACGAACTCCCTTTAGTGAGGGTTAAT-3'
GLI	45	5'-TAATACGACTCACTATAGGGTCATTTCACAATTCAATTACTCAACTCTAGTGCCTAAAGAGTAG -3'<br>5'-PO4-GGAATCTCATCCATCACAGATCCCTTTAGTGAGGGTTAAT-3'
SERPINE1	46	5'TAATACGACTCACTATAGGGTACATCAACAATTCATTCAATACACCTACCAGCCAGAGGGTAGG-3'<br>5'-PO4-GCACAAAGATGGATGTAATGTCCCTTTAGTGAGGGTTAAT-3'
SSX3	47	5'-TAATACGACTCACTATAGGGCTTCTCATTAACTTACTTCATAATAAGAGATCAGCGATCCTGAG-3'<br>5'-PO4-GAAGATGATGAGTAACTCCCTCCCTTTAGTGAGGGTTAAT-3'
PDGFA	48	5'-TAATACGACTCACTATAGGG-AAACAAACTTCACATCTCAATAATCGCAGTGCACACCTAGAATG-3'<br>5'-PO4-CTGTGTTCCCACGCGGCACGTCCCTTTAGTGAGGGTTAAT-3'
FLI	49	5'-TAATACGACTCACTATAGGGTCATCAATCTTTCAATTTACTTACTCCTCCATGCCTGTCACTTC-3'<br>5'-PO4-CTCCAGCTTCTTTGGAGCCGTCCCTTTAGTGAGGGTTAAT-3'
