# Supplementary material for: Signature-Based Small Molecule Screening Identifies Cytosine Arabinoside as an EWS/FLI Modulator in Ewing Sarcoma
Source: PLoS Med. 2007 Apr 10;4(4):e122. doi: 10.1371/journal.pmed.0040122 (PMC1851624; doi:10.1371/journal.pmed.0040122)
Supplement: Supplementary file 6 [file pmed.0040122.st002.pdf]

Supplementary Table 2

Gene sets used in GSEA

Up in RNAi and Sign Confirmed in Inducible

Probe Set ID	Accession	Gene Symbol	Gene Title
200660_at	NM_005620	S100A11	S100 calcium binding protein A11 (calgizzarin)
200785_s_at	NM_002332	LRP1	low density lipoprotein-related protein 1 (alpha-2-macroglobulin receptor)
201325_s_at	NM_001423	EMP1	epithelial membrane protein 1
201373_at	NM_000445	PLEC1	plectin 1, intermediate filament binding protein 500kDa
201466_s_at	NM_002228	JUN	v-jun sarcoma virus 17 oncogene homolog (avian)
201952_at	AA156721	ALCAM	activated leukocyte cell adhesion molecule
202042_at	NM_002109	HARS	histidyl-tRNA synthetase
202074_s_at	NM_021980	OPTN	optineurin
202087_s_at	NM_001912	CTSL	cathepsin L
202588_at	NM_000476	AK1	adenylate kinase 1
202609_at	NM_004447	EPS8	epidermal growth factor receptor pathway substrate 8
202733_at	NM_004199	P4HA2	procollagen-proline, 2-oxoglutarate 4-dioxygenase (proline 4-hydroxylase), alpha polypeptide II
202756_s_at	NM_002081	GPC1	glypican 1
202786_at	NM_013233	STK39	serine threonine kinase 39 (STE20/SPS1 homolog, yeast)
202859_x_at	NM_000584	IL8	interleukin 8
203083_at	NM_003247	THBS2	thrombospondin 2
203186_s_at	NM_002961	S100A4	S100 calcium binding protein A4 (calcium protein, calvasculin, metastasin, murine placental homolog)
203232_s_at	NM_000332	ATXN1	ataxin 1
203233_at	NM_000418	IL4R	interleukin 4 receptor
203771_s_at	AA740186	BLVRA	biliverdin reductase A
203821_at	NM_001945	HBEGF	heparin-binding EGF-like growth factor
203939_at	NM_002526	NT5E	5'-nucleotidase, ecto (CD73)
203955_at	NM_014811	KIAA0649	KIAA0649
204011_at	NM_005842	SPRY2	sprouty homolog 2 (Drosophila)
204472_at	NM_005261	GEM	GTP binding protein overexpressed in skeletal muscle
204518_s_at	NM_000943	PPIC	peptidylprolyl isomerase C (cyclophilin C)
204602_at	NM_012242	DKK1	dickkopf homolog 1 (Xenopus laevis)
204818_at	NM_002153	HSD17B2	hydroxysteroid (17-beta) dehydrogenase 2
205259_at	NM_000901	NR3C2	nuclear receptor subfamily 3, group C, member 2
205479_s_at	NM_002658	PLAU	plasminogen activator, urokinase
205574_x_at	NM_001199	BMP1	bone morphogenetic protein 1
206157_at	NM_002852	PTX3	pentraxin-related gene, rapidly induced by IL-1 beta
206375_s_at	NM_006308	HSPB3	heat shock 27kDa protein 3
206805_at	NM_006080	SEMA3A	sema domain, immunoglobulin domain (Ig), short basic domain, secreted, (semaphorin) 3A
207595_s_at	NM_006132	BMP1	bone morphogenetic protein 1
207765_s_at	NM_025182	KIAA1539	KIAA1539
208960_s_at	BE675435	KLF6	Kruppel-like factor 6
208961_s_at	AB017493	KLF6	Kruppel-like factor 6
209101_at	M92934	CTGF	connective tissue growth factor
209195_s_at	AF250226	ADCY6	adenylate cyclase 6
209276_s_at	AF162769	GLRX	glutaredoxin (thioltransferase)
209652_s_at	BC001422	PGF	placental growth factor, vascular endothelial growth factor-related protein
209834_at	AB017915	CHST3	carbohydrate (chondroitin 6) sulfotransferase 3
210517_s_at	AB003476	AKAP12	A kinase (PRKA) anchor protein (gravin) 12
210540_s_at	BC004523	B4GALT4	UDP-Gal:betaGlcNAc beta 1,4- galactosyltransferase, polypeptide 4
210592_s_at	M55580	SAT	spermidine/spermine N1-acetyltransferase

212089_at	M13452	LMNA	lamin A/C
212347_x_at	AA831438	MXD4	MAX dimerization protein 4
212473_s_at	BE965029	MICAL2	microtubule associated monoxygenase, calponin and LIM domain containing 2
212923_s_at	AK024828	C6orf145	chromosome 6 open reading frame 145
213416_at	BG532690	ITGA4	integrin, alpha 4 (antigen CD49D, alpha 4 subunit of VLA-4 receptor)
213455_at	W87466	LOC283677	hypothetical LOC283677
213462_at	AW000928	NPAS2	neuronal PAS domain protein 2
213727_x_at	AI743654	MPPE1	metallophosphoesterase 1
214110_s_at	BF195104	LOC440886	Similar to lymphocyte-specific protein 1
217983_s_at	NM_003730	RNASET2	ribonuclease T2
218246_at	NM_024544	C1orf166	chromosome 1 open reading frame 166
218880_at	N36408	FOSL2	FOS-like antigen 2
219410_at	NM_018004	TMEM45A	transmembrane protein 45A
220974_x_at	NM_030971	SFXN3	sideroflexin 3 /// sideroflexin 3
221031_s_at	NM_030817	DKFZP434F0:	hypothetical protein DKFZp434F0318 /// hypothetical protein DKFZp434F0318
221667_s_at	AF133207	HSPB8	heat shock 22kDa protein 8
221710_x_at	BC006241	C1orf78	chromosome 1 open reading frame 78 /// chromosome 1 open reading frame 78
221779_at	BC001090	MICAL-L1	MICAL-like 1
221805_at	AL537457	NEFL	neurofilament, light polypeptide 68kDa
221816_s_at	BF055474	PHF11	PHD finger protein 11

Down in RNAi and Sign Confirmed in Inducible

Probe Set ID	Accession	Gene Symbol	Gene Title
202747_s_at	NM_004867	ITM2A	integral membrane protein 2A
203139_at	NM_004938	DAPK1	death-associated protein kinase 1
203323_at	BF197655	CAV2	caveolin 2
204149_s_at	NM_000850	GSTM4	glutathione S-transferase M4
204165_at	NM_003931	WASF1	WAS protein family, member 1
204393_s_at	NM_001099	ACPP	acid phosphatase, prostate
204457_s_at	NM_002048	GAS1	growth arrest-specific 1
204600_at	NM_004443	EPHB3	EPH receptor B3
204735_at	NM_006202	PDE4A	phosphodiesterase 4A, cAMP-specific (phosphodiesterase E2 dunce homolog, Drosophila)
205049_s_at	NM_001783	CD79A	CD79A antigen (immunoglobulin-associated alpha) / CD79A antigen (immunoglobulin-associated alpha)
205227_at	NM_002182	IL1RAP	interleukin 1 receptor accessory protein
205440_s_at	NM_000909	NPY1R	neuropeptide Y receptor Y1
205656_at	NM_014459	PCDH17	protocadherin 17
205728_at	AL022718	---	CDNA clone IMAGE:4811759
205770_at	NM_000637	GSR	glutathione reductase
205771_s_at	AL137063	AKAP7	A kinase (PRKA) anchor protein 7
205826_at	NM_003970	MYOM2	myomesin (M-protein) 2, 165kDa /// myomesin (M-protein) 2, 165kDa
205827_at	NM_000729	CCK	cholecystokinin
205879_x_at	BC004257	RET	ret proto-oncogene (multiple endocrine neoplasia and medullary thyroid carcinoma 1)
206045_s_at	NM_003787	NOL4	nucleolar protein 4
206191_at	NM_001248	ENTPD3	ectonucleoside triphosphate diphosphohydrolase 3
206253_at	NM_001364	DLG2	discs, large homolog 2, chapsyn-110 (Drosophila)
206315_at	NM_004750	CRLF1	cytokine receptor-like factor 1
206626_x_at	BC001003	SSX1	synovial sarcoma, X breakpoint 1
206645_s_at	NM_000475	NR0B1	nuclear receptor subfamily 0, group B, member 1
206646_at	NM_005269	GLI1	glioma-associated oncogene homolog 1 (zinc finger protein)
206812_at	NM_000025	ADRB3	adrenergic, beta-3-, receptor

206915_at	NM_002509	NKX2-2	NK2 transcription factor related, locus 2 (Drosophila)
207397_s_at	NM_000523	HOXD13	homeo box D13
207493_x_at	NM_003147	SSX2	synovial sarcoma, X breakpoint 2
207666_x_at	NM_021014	SSX3	synovial sarcoma, X breakpoint 3
207957_s_at	NM_002738	ATP6AP1	ATPase, H+ transporting, lysosomal accessory protein 1
208345_s_at	NM_002699	POU3F1	POU domain, class 3, transcription factor 1
209242_at	AL042588	PEG3	paternally expressed 3
209243_s_at	AF208967	PEG3	paternally expressed 3
209616_s_at	S73751	CES1	carboxylesterase 1 (monocyte/macrophage serine esterase 1)
209683_at	AA243659	FAM49A	Family with sequence similarity 49, member A
209685_s_at	M13975	PRKCB1	protein kinase C, beta 1
210084_x_at	AF206665	TPSAB1	tryptase alpha/beta 1
210375_at	X83858	PTGER3	prostaglandin E receptor 3 (subtype EP3)
210394_x_at	BC005325	SSX4	synovial sarcoma, X breakpoint 4
210831_s_at	L27489	PTGER3	prostaglandin E receptor 3 (subtype EP3)
210832_x_at	D38298	PTGER3	prostaglandin E receptor 3 (subtype EP3)
210963_s_at	U94363	GYG2	glycogenin 2
210964_s_at	U94364	GYG2	glycogenin 2
211670_x_at	S82471	SSX3	synovial sarcoma, X breakpoint 3 /// synovial sarcoma, X breakpoint 3
211709_s_at	BC005810	CLEC11A	C-type lectin domain family 11, member A /// C-type lectin domain family 11, member A
211825_s_at	AF327066	FLI1	Friend leukemia virus integration 1
212670_at	AA479278	ELN	elastin (supravalvular aortic stenosis, Williams-Beuren syndrome)
212738_at	AV717623	ARHGAP19	Rho GTPase activating protein 19
213707_s_at	NM_005221	DLX5	distal-less homeo box 5
214754_at	AB007861	MGC22014	hypothetical protein MGC22014
214881_s_at	X56687	UBTF	upstream binding transcription factor, RNA polymerase I
215695_s_at	U94357	GYG2	glycogenin 2
216052_x_at	AF115765	ARTN	artemin
216474_x_at	AF206667	TPSAB1	tryptase alpha/beta 1
216623_x_at	AK025084	TNRC9	trinucleotide repeat containing 9
218445_at	NM_018649	H2AFY2	H2A histone family, member Y2
218563_at	NM_004542	NDUFA3	NADH dehydrogenase (ubiquinone) 1 alpha subcomplex, 3, 9kDa
218825_at	NM_016215	EGFL7	EGF-like-domain, multiple 7
218831_s_at	NM_004107	FCGRT	Fc fragment of IgG, receptor, transporter, alpha
218976_at	NM_021800	DNAJC12	DnaJ (Hsp40) homolog, subfamily C, member 12
219090_at	NM_020689	SLC24A3	solute carrier family 24 (sodium/potassium/calcium exchanger), member 3
219300_s_at	AB020675	CNTNAP2	contactin associated protein-like 2
219301_s_at	AU144598	CNTNAP2	contactin associated protein-like 2
219414_at	NM_022131	CLSTN2	calsyntenin 2
219471_at	NM_025113	C13orf18	chromosome 13 open reading frame 18
219745_at	NM_024789	C10orf77	chromosome 10 open reading frame 77
219976_at	NM_015888	HOOK1	hook homolog 1 (Drosophila)
220462_at	NM_024969	TAIP-2	TGF-beta induced apotosis protein 2
221011_s_at	NM_030915	LBH	likely ortholog of mouse limb-bud and heart gene /// likely ortholog of mouse limb-bud and heart gene
221147_x_at	NM_018560	WWOX	WW domain containing oxidoreductase
221606_s_at	BC005342	NSBP1	nucleosomal binding protein 1
39966_at	AF059274	CSPG5	chondroitin sulfate proteoglycan 5 (neuroglycan C)
44790_s_at	AI129310	C13orf18	chromosome 13 open reading frame 18
47560_at	AI525402	LPHN1	latrophilin 1
57588_at	R62432	SLC24A3	solute carrier family 24 (sodium/potassium/calcium exchanger), member 3
