# Supplementary material for: Signature-Based Small Molecule Screening Identifies Cytosine Arabinoside as an EWS/FLI Modulator in Ewing Sarcoma
Source: PLoS Med. 2007 Apr 10;4(4):e122. doi: 10.1371/journal.pmed.0040122 (PMC1851624; doi:10.1371/journal.pmed.0040122)
Supplement: Supplementary file 7 [file pmed.0040122.st003.pdf]

Supplementary Table 3
GSEA results for the 66 gene EWS/FLI knockdown signature

Compound	Concentration	# of Samples	P-Value	Enrichment Score
ARA-C	170 nM (EC50)	6	0.040381793	-0.5694869
ARA-C	340 nM (2x EC50)	6	7.45E-04	-0.73430395
Doxorubicin	60 nM (EC50)	6	0.013483146	-0.6324752
Doxorubicin	120 nM (2x EC50)	5	0.016988417	-0.6220424
Puromycin	90 nM (EC50)	5	0.064150944	0.45456073
Puromycin	180 nM (2x EC50)	3	0.09796807	0.43447396
