# Supplementary material for: Signature-Based Small Molecule Screening Identifies Cytosine Arabinoside as an EWS/FLI Modulator in Ewing Sarcoma
Source: PLoS Med. 2007 Apr 10;4(4):e122. doi: 10.1371/journal.pmed.0040122 (PMC1851624; doi:10.1371/journal.pmed.0040122)
Supplement: Supplementary file 8 [file pmed.0040122.st004.pdf]

Supplementary Table 4

Affymetrix U133A Ewing Samples

Raw data files can be located at www.broad.mit.edu/cancer/Ewing_ARA-C

Complete Sample Tag	Scan Name	Treatment	Concentratior	Duration	Archive File
A673_24hr_1.A01	5202764005789147112904.A01	DMSO		24 Hours	
A673_24hr_2.A02	5202764005789147112904.A02	DMSO		24 Hours	
A673_24hr_3.A03	5202764005789147112904.A03	DMSO		24 Hours	
A673_24hr_4.G02	5202764005789147112904.G02	DMSO		24 Hours	
A673_24hr_5.G03	5202764005789147112904.G03	DMSO		24 Hours	
A673_24hr_6.G04	5202764005789147112904.G04	DMSO		24 Hours	
A673_D3_1.C01	5202764005789147112904.C01	DMSO		3 Days	
A673_D3_2.C02	5202764005789147112904.C02	DMSO		3 Days	
A673_D3_4.G10	5202764005789147112904.G10	DMSO		3 Days	Ewings_Control_CELFiles_060510.zip
A673_D3_5.G11	5202764005789147112904.G11	DMSO		3 Days	
A673_D3_6.G12	5202764005789147112904.G12	DMSO		3 Days	
A673_D5_1.E01	5202764005789147112904.E01	DMSO		5 Days	
A673_D5_2.E02	5202764005789147112904.E02	DMSO		5 Days	
A673_D5_3.E03	5202764005789147112904.E03	DMSO		5 Days	
A673_D5_4.H02	5202764005789147112904.H02	DMSO		5 Days	
A673_D5_5.H03	5202764005789147112904.H03	DMSO		5 Days	
A673_D5_6.H04	5202764005789147112904.H04	DMSO		5 Days	
AraC_0p17uM_24hr_1.A04	5202764005789147112904.A04	ARA-C	0.17 uM	24 Hours	
AraC_0p17uM_24hr_2.A05	5202764005789147112904.A05	ARA-C	0.17 uM	24 Hours	
AraC_0p17uM_24hr_3.A06	5202764005789147112904.A06	ARA-C	0.17 uM	24 Hours	
AraC_0p17uM_D3_1.C04	5202764005789147112904.C04	ARA-C	0.17 uM	3 Days	
AraC_0p17uM_D3_2.C05	5202764005789147112904.C05	ARA-C	0.17 uM	3 Days	
AraC_0p17uM_D3_3.C06	5202764005789147112904.C06	ARA-C	0.17 uM	3 Days	
AraC_0p17uM_D5_1.E04	5202764005789147112904.E04	ARA-C	0.17 uM	5 Days	
AraC_0p17uM_D5_2.E05	5202764005789147112904.E05	ARA-C	0.17 uM	5 Days	
AraC_0p17uM_D5_3.E06	5202764005789147112904.E06	ARA-C	0.17 uM	5 Days	Ewings_ARA-C_CELFiles_060510.zip
AraC_0p34uM_24h_1.G05	5202764005789147112904.G05	ARA-C	0.34 uM	24 Hours	
AraC_0p34uM_24h_2.G06	5202764005789147112904.G06	ARA-C	0.34 uM	24 Hours	
AraC_0p34uM_24h_3.G08	5202764005789147112904.G08	ARA-C	0.34 uM	24 Hours	
AraC_0p34uM_D3_1.H07	5202764005789147112904.H07	ARA-C	0.34 uM	3 Days	
AraC_0p34uM_D3_2.H08	5202764005789147112904.H08	ARA-C	0.34 uM	3 Days	
AraC_0p34uM_D3_3.H01	5202764005789147112904.H01	ARA-C	0.34 uM	3 Days	
AraC_0p34uM_D5_1.H05	5202764005789147112904.H05	ARA-C	0.34 uM	5 Days	
AraC_0p34uM_D5_2.H06	5202764005789147112904.H06	ARA-C	0.34 uM	5 Days	
AraC_0p34uM_D5_3.H09	5202764005789147112904.H09	ARA-C	0.34 uM	5 Days	
Doxo_59p88nM_24hr_1.A10	5202764005789147112904.A10	Doxorubicin	59.88 nM	24 Hours	
Doxo_59p88nM_24hr_2.A11	5202764005789147112904.A11	Doxorubicin	59.88 nM	24 Hours	
Doxo_59p88nM_24hr_3.A12	5202764005789147112904.A12	Doxorubicin	59.88 nM	24 Hours	
Doxo_59p88nM_D3_1.C10	5202764005789147112904.C10	Doxorubicin	59.88 nM	3 Days	
Doxo_59p88nM_D3_2.C11	5202764005789147112904.C11	Doxorubicin	59.88 nM	3 Days	
Doxo_59p88nM_D3_3.D07	5202764005789147112904.D07	Doxorubicin	59.88 nM	3 Days	
Doxo_59p88nM_D5_1.E09	5202764005789147112904.E09	Doxorubicin	59.88 nM	5 Days	
Doxo_59p88nM_D5_2.E11	5202764005789147112904.E11	Doxorubicin	59.88 nM	5 Days	
Doxo_59p88nM_D5_3.E12	5202764005789147112904.E12	Doxorubicin	59.88 nM	5 Days	Ewings_Dox_CELFiles_060510.zip
Doxo_119p76nM_24hr_1.B01	5202764005789147112904.B01	Doxorubicin	119.76 nM	24 Hours	
Doxo_119p76nM_24hr_2.B02	5202764005789147112904.B02	Doxorubicin	119.76 nM	24 Hours	
Doxo_119p76nM_24hr_3.B03	5202764005789147112904.B03	Doxorubicin	119.76 nM	24 Hours	

Doxo_119p76nM_D3_1.D01	5202764005789147112904.D01	Doxorubicin	119.76 nM	3 Days	
Doxo_119p76nM_D3_2.D02	5202764005789147112904.D02	Doxorubicin	119.76 nM	3 Days	
Doxo_119p76nM_D3_3.D03	5202764005789147112904.D03	Doxorubicin	119.76 nM	3 Days	
Doxo_119p76nM_D5_1.F01	5202764005789147112904.F01	Doxorubicin	119.76 nM	5 Days	
Doxo_119p76nM_D5_3.F03	5202764005789147112904.F03	Doxorubicin	119.76 nM	5 Days	
Puro_0p09uM_24hr_1.B07	5202764005789147112904.B07	Puromycin	0.09 uM	24 Hours	
Puro_0p09uM_24hr_2.B08	5202764005789147112904.B08	Puromycin	0.09 uM	24 Hours	
Puro_0p09uM_24hr_3.B09	5202764005789147112904.B09	Puromycin	0.09 uM	24 Hours	
Puro_0p09uM_D3_1.D09	5202764005789147112904.D09	Puromycin	0.09 uM	3 Days	
Puro_0p09uM_D3_2.D10	5202764005789147112904.D10	Puromycin	0.09 uM	3 Days	
Puro_0p09uM_D3_3.D11	5202764005789147112904.D11	Puromycin	0.09 uM	3 Days	
Puro_0p09uM_D5_1.F08	5202764005789147112904.F08	Puromycin	0.09 uM	5 Days	
Puro_0p09uM_D5_2.F09	5202764005789147112904.F09	Puromycin	0.09 uM	5 Days	Ewings_Puro_CELFiles_060510.zip
Puro_0p09uM_D5_3.F10	5202764005789147112904.F10	Puromycin	0.09 uM	5 Days	
Puro_0p18uM_24hr_1.B10	5202764005789147112904.B10	Puromycin	0.18 uM	24 Hours	
Puro_0p18uM_24hr_2.B11	5202764005789147112904.B11	Puromycin	0.18 uM	24 Hours	
Puro_0p18uM_24hr_3.B12	5202764005789147112904.B12	Puromycin	0.18 uM	24 Hours	
Puro_0p18uM_D3_1.D12	5202764005789147112904.D12	Puromycin	0.18 uM	3 Days	
Puro_0p18uM_D3_2.E07	5202764005789147112904.E07	Puromycin	0.18 uM	3 Days	
Puro_0p18uM_D3_3.E08	5202764005789147112904.E08	Puromycin	0.18 uM	3 Days	
Puro_0p18uM_D5_3.G07	5202764005789147112904.G07	Puromycin	0.18 uM	5 Days	
