# Supplementary material for: Signature-Based Small Molecule Screening Identifies Cytosine Arabinoside as an EWS/FLI Modulator in Ewing Sarcoma
Source: PLoS Med. 2007 Apr 10;4(4):e122. doi: 10.1371/journal.pmed.0040122 (PMC1851624; doi:10.1371/journal.pmed.0040122)
Supplement: Supplementary file 9 [file pmed.0040122.st005.pdf]

Supplementary Table 5

Cell Line	Day 3 EC50 μM	Day 6 EC50 μM
**Ewing Cell Lines**		
A673	0.17	0.05
EWS502	0.38	0.28
TC32	0.08	0.06
TC71	0.10	0.07
**Mean**	**0.18**	**0.11**
**Carcinoma Cell Lines**		
A549	0.53	0.58
HCT116	0.25	0.09
SW480	15.10	1.06
T47D	40.80	6.67
**Mean**	**14.17**	**2.10**
